# The profound implications of mitochondrial myopathy on activities of daily living: an observational qualitative study of standardized structured and semi-structured patient interviews

**DOI:** 10.1177/20406223251344763

**Published:** 2025-07-25

**Authors:** Elizabeth M. McCormick, James T. Peterson, Joaquim Diego D. Santos, Jean Flickinger, Rui Xiao, Richard Haas, Zarazuela Zolkipli-Cunningham

**Affiliations:** Mitochondrial Medicine Frontier Program, Division of Human Genetics, Department of Pediatrics, The Children’s Hospital of Philadelphia, Philadelphia, PA, USA; Mitochondrial Medicine Frontier Program, Division of Human Genetics, Department of Pediatrics, The Children’s Hospital of Philadelphia, Philadelphia, PA, USA; Mitochondrial Medicine Frontier Program, Division of Human Genetics, Department of Pediatrics, The Children’s Hospital of Philadelphia, Philadelphia, PA, USA; Mitochondrial Medicine Frontier Program, Division of Human Genetics, Department of Pediatrics, The Children’s Hospital of Philadelphia, Philadelphia, PA, USA; Center for Rehabilitation, Children’s Hospital of Philadelphia, Philadelphia, PA, USA; Department of Biostatistics, Epidemiology and Informatics, University of Pennsylvania Perelman School of Medicine, Philadelphia, PA, USA; Department of Pediatrics, University of Pennsylvania Perelman School of Medicine, Philadelphia, PA, USA; Department of Neurosciences, University of California—San Diego, San Diego, CA, USA; Department of Pediatrics, University of California—San Diego, San Diego, CA, USA; Division of Neurology, Rady Children’s Hospital, San Diego, CA, USA; Mitochondrial Medicine Frontier Program, Division of Human Genetics, Department of Pediatrics, The Children’s Hospital of Philadelphia, 3500 Civic Center Blvd, Philadelphia, PA 19104, USA; Department of Pediatrics, University of Pennsylvania Perelman School of Medicine, Philadelphia, PA, USA

**Keywords:** Mitochondrial Myopathy (MM), activities of daily living (ADLs), adaptation, impact, muscle weakness, muscle fatigue, exercise intolerance, imbalance, dexterity

## Abstract

**Background:**

The impact of Mitochondrial Myopathy (MM) symptoms on functional ability across activities of daily living (ADLs) has not been fully characterized, nor is it understood how MM patients define their key symptoms. Furthermore, it is unclear what MM individuals perceive as a clinically meaningful improvement.

**Objective:**

We sought to characterize how MM patients feel about their symptoms in the key MM domains of muscle weakness, muscle fatigue, exercise intolerance, imbalance, and peripheral neuropathy; as well as their functional ability.

**Design:**

We conducted a single-center, observational, qualitative study that involved standardized structured and semi-structured patient interviews.

**Methods:**

Most interview questions were open-ended, allowing individuals to provide personalized narratives that were transcribed in real time. A total of 33 individuals with MM were interviewed either in-person or remotely. Interview transcripts underwent thematic analysis in accordance with grounded theory. Data was presented using a mixed-methods approach.

**Results:**

Subjects provided extensive narratives that demonstrated the substantial and widespread impact of MM across many aspects of MM patient lives, including the impact of each MM domain of muscle weakness, muscle fatigue, exercise intolerance, imbalance, and peripheral neuropathy on ADLs; the need to adapt to preserve independence and quality of life (QOL); impaired self-perception, participation in social activities, hobbies, and relationships; and change in circumstances over time.

**Conclusion:**

These meaningful insights highlight the critical and emergent need for approved drug treatment(s) in this profoundly burdened patient population. Our results will serve as a comprehensive resource to inform the physician, patient, industry and advocacy communities on outcome measure selection and clinical trial design; and to help inform regulatory agencies in the United States Food and Drug Administration (FDA) drug approval process for MM.

## Background

Primary mitochondrial disease (PMD) encompasses a heterogeneous group of genetic disorders with varying but typically multi-organ system involvement that affects approximately 1 in 4300 individuals.^1^ PMD is caused by pathogenic variants in more than 425 genes in the nuclear or mitochondrial genomes.^2,3^ Affected individuals report a mean of 16 symptoms that include muscle weakness, fatigue, exercise intolerance, and imbalance as the most commonly reported,^4^ and in addition to gastrointestinal (GI) problems, were the top five reported symptoms to encourage clinical trial participation in a PMD cohort (*n* = 290).^4^ In addition, peripheral neuropathy may occur in up to one-third of PMD patients and is often missed due to the more prominent symptoms of a multi-system disorder.^5^

Several studies have reported the substantial burden of the physical symptoms of PMD on quality of life (QOL), utilizing patient^6–12^ and/or caregiver-reported outcome measures.^13,14^ Impaired physical function, mobility, and fatigue contribute to loss of independence and decreased QOL in children and adults with PMD.^6,11,15^ A further cause of decline in QOL is the high hospitalization rates associated with serious illnesses, prolonged stays, and substantial costs^16,17^ that collectively add to the considerable and global burden of PMD. In addition, there is a high in-hospital mortality rate with a ~6-fold increase in children and ~3-fold increase in adults in the United States (US) when compared to individuals without PMD.^17^ Indeed, the high economic burden of PMD has been reported across the United States,^18^ Canada,^16^ and Australia.^19^

Despite the significant physical, emotional, and socioeconomic burden of PMD, there are currently no Food and Drug Administration (FDA) approved therapies in the United States.^20^ However, numerous drug intervention clinical trials have emerged for PMD,^21,22^ and specifically in Mitochondrial Myopathy (MM).^23–26^ Individuals with MM, a subset of PMD, have predominant symptoms of myopathy.^27^ The breadth of motor function impairments in MM have been demonstrated in Mitochondrial Myopathy Composite Assessment Tool (MM-COAST) objective assessments.^27^ Individuals with MM are known to desire improvements in social and leisure activities, and work/occupations that are limited by their MM diagnosis.^28^ However, deeper understanding of the consequences of having symptoms in each of the key MM domains^27^ of muscle weakness, muscle fatigue, exercise intolerance, imbalance, and peripheral neuropathy on activities of daily life (ADLs) and independent functional ability in MM individuals is lacking. While studies have shown that many children with PMD perform ADLs as their healthy peers do, they differ in the amount of support required to complete ADLs.^29^

Qualitative studies reporting patient and/or caregiver perspectives of the impact of MM on ADLs are limited, as compared to existing literature in other rare disorders, including Ehlers Danlos syndrome^30^ and Spinal Muscular Atrophy.^31^ The quantitative results of our survey study of 290 self-reported PMD subjects successfully demonstrated the most common symptoms experienced and would motivate clinical trial participation.^4^ However, patient/caregiver surveys do not holistically illustrate the profound, multidimensional impact of having a broad range of chronic MM symptoms on the daily lives of MM individuals. Insights and perspectives disclosed at qualitative patient interviews are far more comprehensive as compared to quantitative survey results with predetermined responses.^32,33^

## Objectives

We conducted a single center, prospective, qualitative study involving standardized semi-structured and structured MM patient and/or caregiver interviews (*n* = 33), to (a) comprehensively characterize the MM patient perspective (i) across their experienced symptom(s) of having an MM diagnosis and (ii) within each individual MM domain of muscle weakness, muscle fatigue, exercise intolerance, and imbalance as identified in our published survey,^4^ as well as peripheral neuropathy that is increasingly observed in MM,^5^ and (b) to characterize the impact of having MM symptoms and their expectations of what a meaningful change would be; building upon the results of our survey study.^4^ The overarching goal of administering open-ended interviews was to harness patient-centered qualitative research methods in order to demonstrate the depth of MM disease burden described in the subject’s own words of how they function and feel due to having a broad range of symptom(s) across their MM diagnosis and within each MM domain.

## Methods and design

### Demographics

Subjects with MM were recruited from the Children’s Hospital of Philadelphia (CHOP) Mitochondrial Medicine Frontier Program and enrolled in CHOP IRB-approved research study #16-013364. Subjects with definite MM caused by pathogenic variant(s) in a nuclear or mitochondrial gene, or those highly suspected to have MM based on clinical and biochemical evidence, and were willing to participate, were enrolled in this study. All participants (or legally authorized representatives of minors or those with diminished capacity) provided written informed consent prior to participating.

### Structured and semi-structured interviews

**Figure 1.**
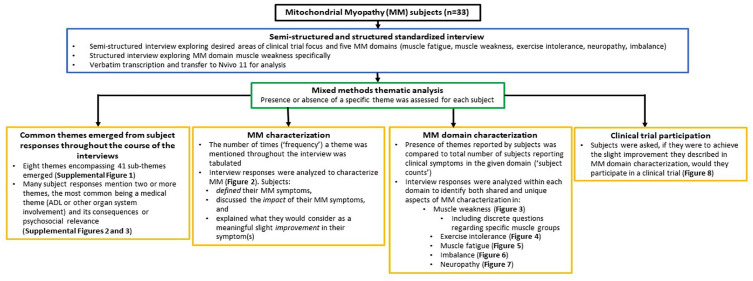
Study overview. Interview transcripts were reviewed for recurrent patterns or topics that were grouped into themes. The number of times a theme was mentioned (“frequency”), or the number of subjects who mentioned a theme (“subject counts”), were quantified by content analysis. Results are presented using mixed methods thematic analysis. ADL, activities of daily life; MM, Mitochondrial Myopathy.

Adult subjects with MM or parents of children with MM (if the affected individual was under the age of 18 or had diminished capacity) participated in structured and semi-structured, standardized interviews (Figure 1). Interview questions were developed with guidance from experts experienced in administering and developing qualitative interviews. Open- and closed-ended questions were included. Questions were designed to elicit unbiased information on (1) the most commonly reported symptoms in the key domains of MM (muscle weakness, muscle fatigue, exercise intolerance, and imbalance as identified in our published survey,^4^ as well as peripheral neuropathy that is increasingly recognized in MM^5^); and (2) other systemic symptoms that subjects reported to be relevant. Once written informed consent was obtained, interviews were conducted by one of three research assistants remotely or in person during routine outpatient clinic visits. Interviews were performed between September 2017 and March 2018. If a subject was <18 years (*n* = 13) or had reduced cognitive or physical ability to participate (*n* = 2), a parent/caregiver participated in the interview, and the individual with MM was encouraged to engage in the conversation when possible.

The semi-structured interview (Supplemental File 1) began with subjects being asked which symptoms should be addressed in a clinical trial, to avoid question-order bias. Subjects reporting exercise intolerance, muscle fatigue, imbalance, and/or peripheral neuropathy were then asked to describe their experienced symptoms (to evaluate the definition of their symptoms in each domain), whereas subjects with muscle weakness were asked structured questions to assess the degree and pattern of muscle group involvement (to understand its effect on daily life). Subjects who reported the presence of symptoms in each domain were then asked to describe how each symptom impacted their daily life, what they considered to be a meaningful slight improvement, and if they would participate in a clinical trial to achieve this meaningful change. Subjects were allowed to freely elaborate their responses and were provided the opportunity to discuss any additional symptoms and issues at the end of the interview. On completion of the interview, subjects were asked if any emotional burden had been incurred. Interview questions were initially piloted in seven subjects. Interview responses were transcribed verbatim in real-time into a Research Electronic Data Capture (REDCap) instrument and de-identified. The study was conducted and reported in accordance with the COnsolidated criteria for REporting Qualitative research (COREQ) statement.^34^

### Coding and thematic analysis

A codebook (Supplemental File 2) was created in a data-driven manner^35^ and in accordance with the grounded theory approach.^36^ Two study team members, who did not administer the interviews, independently coded the interview transcripts in Nvivo 11 software. The coders (E.M.M. and J.T.P.) were trained in psychosocial aspects of genetic diseases as clinical and certified genetic counselors. Transcript analysis review was first performed by one team member (E.M.M.) to identify and group common subject responses. These common responses were considered themes, which were labeled with a short name (the code) to generate the codebook. Codes were sequentially added to the codebook as they arose in subsequent interviews. Codebook and transcripts were then reviewed by a second team member (J.T.P.). In this iterative process, the coders periodically met to assess for the presence of novel themes and to discuss any discrepancies. After completion of 33 subject interviews, no additional themes were identified. Thus, data saturation was reached.^37^ All final coding was compared for inter-rater reliability, and the final codebook was achieved by group consensus (E.M.M., J.T.P.).

The number of subjects who mentioned each symptom and the frequency at which each symptom and/or common theme was mentioned in the discussion were quantified by content analysis. Themes fell into one of two broad categories: medical themes and psychosocial/subjective themes. These themes were initially described in terms of how many subjects mentioned them throughout the interviews, or “subject counts” (Supplemental Figure 1). Co-occurrence of themes was also analyzed, referred to as “frequency of theme co-occurrences,” whereby the number of times a theme was mentioned by any subject throughout the course of the interview in relation to another theme was described (Supplemental Figures 2 and 3).

**Figure 2.**
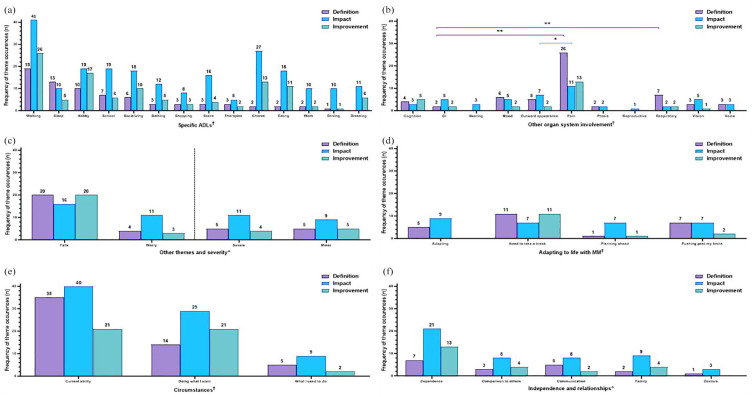
Frequencies of themes being mentioned in response to interview questions. Subjects were asked to define their main symptoms (purple bars), discuss the impact of MM (blue bars), and explain what a meaningful slight improvement would look like (green bars). Results are presented as the number of times (“frequency”) a theme was mentioned throughout the interview. The occurrence of specific themes related to (a) ADLs, (b) other organ system involvement, (c) falls, worry, and severity, (d) adapting to life with MM, (e) circumstances, and (f) independence and relationships are displayed. Color coding of brackets to demonstrate *p*-values corresponds to figure key. ^†^(a, d, e) Pairwise comparisons performed using Fisher’s exact test, *p* ⩾ 0.05, all non-significant. ^†^(b) Pairwise comparisons performed using Fisher’s exact test, *p < 0.05; **p < 0.001. All other pairwise comparisons were non-significant. ^^^(c, f) Statistical analysis was not performed due to low numbers. ADLs, activity of daily living; GI, gastrointestinal involvement; MM, Mitochondrial Myopathy.

**Figure 3.**
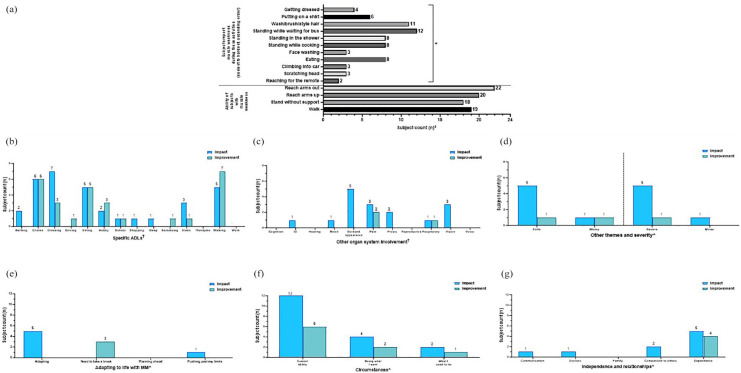
Characterization of muscle weakness displayed by subject counts. Muscle weakness was characterized by (a) discrete questions centered on common daily activities, displayed in ascending order of increasing difficulty (least difficult, lowermost) based on the metabolic equivalent of tasks (METs) and (b-g) presence of specific themes mentioned in open-ended questions on impact (blue bars) and slight improvement (green bars) related to (b) activity of daily living (ADLs), (c) other organ system involvement, (d) falls, worry, and severity, (e) adapting to life with MM, (f) circumstances, and (g) independence and relationships. ^‡^(a) Cochran-Armitage trend test was performed and showed a significant (*p < 0.05) increasing trend in the number of subjects who noted difficulty with increasingly harder tasks (from lowermost to the top). ^†^(b, c) Pairwise comparisons performed using Fisher’s exact test, *p* ⩾ 0.05, all non-significant. ^^^(d–g) Statistical analysis was not performed due to low numbers. ADLs, activity of daily living; GI, gastrointestinal involvement; METs, metabolic equivalents of tasks; MM, Mitochondrial Myopathy.

**Figure 4.**
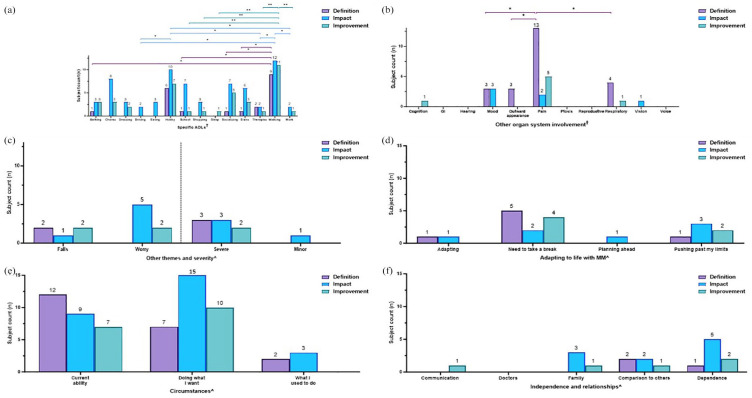
Within-domain analysis for ADL themes noted in subjects with exercise intolerance. Characterization of exercise intolerance: occurrence of specific themes related to (a) ADLs, (b) other organ system involvement, (c) falls, worry, and severity, (d) adapting to life with MM, (e) circumstances, and (f) independence and relationships are displayed. Bars represent the number of subjects reporting each theme (“subject counts”) when asked about the definition of symptoms (purple bars), impact (blue bars), and what a meaningful slight improvement would be (green bars) in each MM domain. Color coding of brackets to demonstrate *p*-values corresponds to the figure key. ^†^(a, b) Pairwise comparisons performed using Fisher’s exact test, *p < 0.05; **p < 0.001. All other pairwise comparisons were non-significant. ^^^(c–f) Statistical analysis was not performed due to low numbers. ADL, activities of daily living; GI, gastrointestinal involvement; MM, Mitochondrial Myopathy.

**Figure 5.**
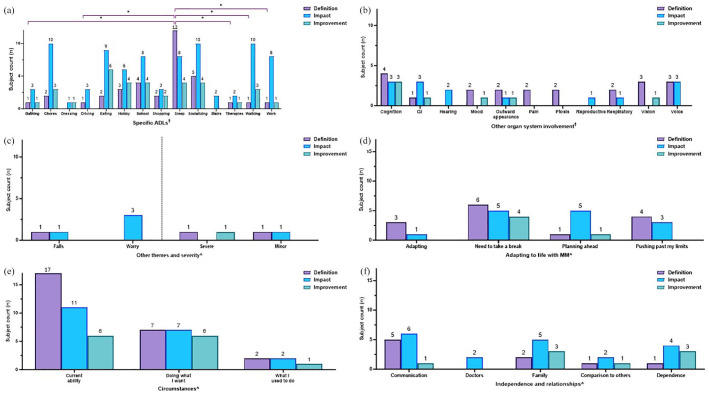
Within-domain analysis for ADL themes noted in subjects with muscle fatigue. Characterization of muscle fatigue: occurrence of specific themes related to (a) ADLs, (b) other organ system involvement, (c) falls, worry, and severity, (d) adapting to life with MM, (e) circumstances, and (f) independence and relationships are displayed. Bars represent the number of subjects reporting each theme (“subject counts”) when asked about the definition of symptoms (purple bars), impact (blue bars), and what a meaningful slight improvement would be (green bars) in each MM domain. Color coding of brackets to demonstrate *p*-values corresponds to figure key. ^†^(a) Pairwise comparisons performed using Fisher’s exact test, *p < 0.05. All other pairwise comparisons were non-significant. ^†^(b) Pairwise comparisons performed using Fisher’s exact test, *p* ⩾ 0.05, all non-significant. ^^^(c–f) Statistical analysis was not performed due to low numbers. ADL, activities of daily living; GI, gastrointestinal involvement; MM, Mitochondrial Myopathy.

**Figure 6.**
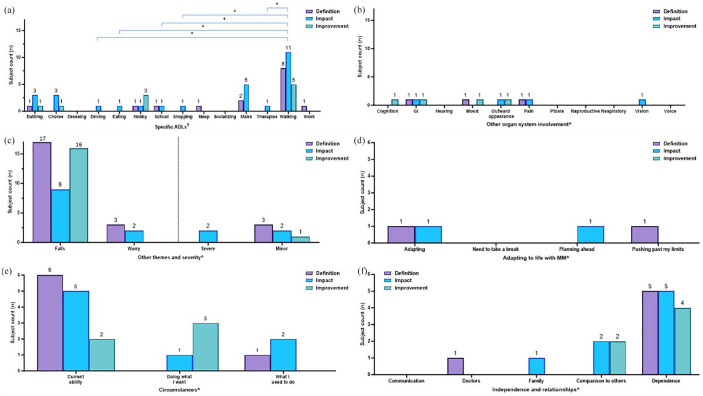
Within-domain analysis for ADL themes noted in subjects with imbalance. Characterization of imbalance: occurrence of specific themes related to (a) ADL, (b) other organ system involvement, (c) falls, worry, and severity, (d) adapting to life with MM, (e) circumstances, and (f) independence and relationships are displayed. Bars represent the number of subjects reporting each theme (“subject counts”) when asked about the definition of symptoms (purple bars), impact (blue bars), and what a meaningful slight improvement would be (green bars) in each MM domain. Color coding of brackets to demonstrate *p*-values corresponds to figure key. ^†^(a) Pairwise comparisons performed using Fisher’s exact test, *p < 0.05. All other pairwise comparisons were non-significant. ^^^(b–f) Statistical analysis was not performed due to low numbers. ADL, activities of daily living; GI, gastrointestinal involvement; MM, Mitochondrial Myopathy.

**Figure 7.**
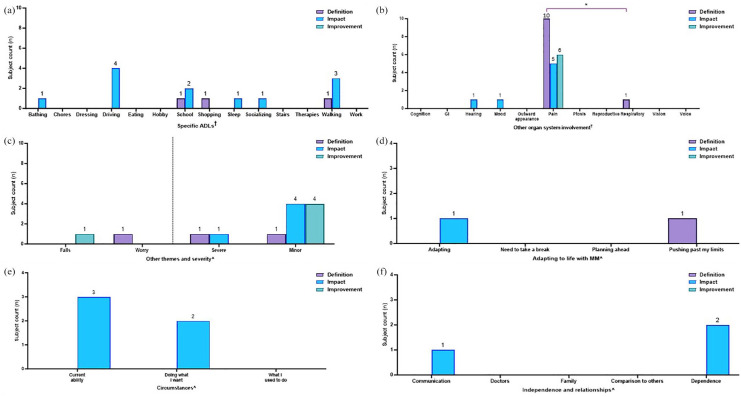
Within-domain analysis for ADL themes noted in subjects with peripheral neuropathy. Characterization of neuropathy: occurrence of specific themes related to (a) ADLs, (b) other organ system involvement, (c) falls, worry, and severity, (d) adapting to life with MM, (e) circumstances, and (f) independence and relationships are displayed. Bars represent the number of subjects reporting each theme (“subject counts”) when asked about the definition of symptoms (purple bars), impact (blue bars), and what a meaningful slight improvement would be (green bars) in each MM domain. Color coding of brackets to demonstrate *p*-values corresponds to figure key. ^†^(a) Pairwise comparisons performed using Fisher’s exact test, *p* ⩾ 0.05, all non-significant. ^†^(b) Pairwise comparisons performed using Fisher’s exact test, *p < 0.05. All other pairwise comparisons were non-significant. ^^^(c–f) Statistical analysis was not performed due to low numbers. ADL, activities of daily living; GI, gastrointestinal involvement; MM, Mitochondrial Myopathy.

The presence of themes reported by subjects were subsequently quantified in terms of (i) overall MM diagnosis characterization (Figure 2) and (ii) MM domain-specific (muscle weakness (Figure 3), exercise intolerance (Figure 4), muscle fatigue (Figure 5), imbalance (Figure 6), and peripheral neuropathy (Figure 7)) characterization.

When considering themes in relation to overall MM diagnosis characterization (Figure 2), results were presented as the cumulative number of times a theme was mentioned throughout the interview, referred to as “frequency” of theme occurrences. Results were not presented as “subject counts” since subjects with symptoms in more than one MM domain may have mentioned a particular theme in one or more domains.

**Figure 8.**
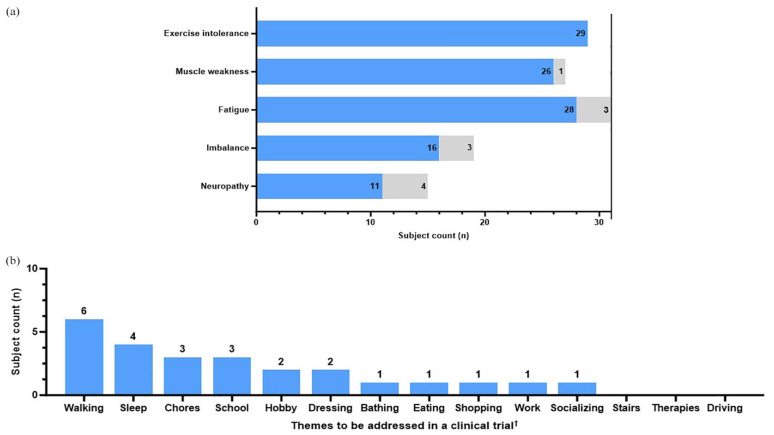
Clinical trial participation. Subjects with symptoms in each MM domain were asked if they would participate in a clinical trial to achieve the “slight improvement” they described in the previous question. (a) Number of subjects in each domain (“subject counts”) who would participate in a clinical trial to achieve their described “slight improvement” (blue) versus those who would not participate (gray). (b) Common themes were discussed when subjects were asked what should be addressed in a clinical trial. ^†^(b) Pairwise comparisons performed using Fisher’s exact test, *p* ⩾ 0.05, non-significant.

By contrast, themes in relation to a specific MM domain (muscle weakness, exercise intolerance, muscle fatigue, imbalance, and peripheral neuropathy) were presented as the number of subjects who mentioned each theme, or “subject counts” (Figures 3–8). If a single theme recurred in one subject’s response, the presence or absence of that specific theme was tabulated as a single count. Responses to questions specifically related to muscle weakness (Figure 3), as well as the desire to participate in clinical trials motivated by symptoms in a given MM domain (Figure 8), were compared to the total number of subjects who reported the corresponding symptoms.

### Data analysis

Data was presented using a mixed-methods approach to display complementarity, where the depth of the MM subject perspectives was demonstrated by qualitative methods and the breadth was displayed by quantitative methods.^38^

Fisher’s exact test was used to compare categorical variables among different groups that were able to be summarized by a contingency table. For data presented as frequencies (Figure 2), the Fisher’s exact test was used to compare the number of times a given theme (e.g., walking) is mentioned in response to the specific question (e.g., definition of muscle weakness) versus the number of mentions in other discussions. As for data presented as theme co-occurrences (Supplemental Figures 2 and 3), the Fisher’s exact test was used to compare the number of times a specific theme was mentioned in relation to another theme versus the number of times a particular theme was mentioned within a different context. For data presented as subject counts (Figures 3–8, Supplemental Figure 1), Fisher’s exact test was used to compare the number of subjects who reported a specific theme (e.g., pain) versus the number of subjects who did not report the given theme across the total cohort who reported the MM domain (e.g., muscle weakness).

The Cochran-Armitage trend test was used to assess the trend in the frequency of ADLs being mentioned in relation to the order of difficulty in conducting the tasks from easiest to hardest. All analyses were conducted in GraphPad Prism (version 10) or RStudio.

### Reporting guidelines

The Strengthening the Reporting of Observational Studies in Epidemiology (STROBE) guidelines for reporting observational studies^39^ were consulted on preparation of the manuscript.

## Results

### Clinical characteristics of study cohort

Thirty-three subjects participated, including 18 adults with clinical symptoms of PMD and 15 parents of individuals (children and adults) with PMD (Table 1). Exemplary quotes from subject interviews are listed in Supplemental Table 1.

**Table 1.** Demographics.

Total subjects, *n* = 33		
Subject relationship to MM	Adults with MM	Parents of child or adult with MM
	18	15
Sex of individual with MM		
Male	7	10
Female	11	5
Age (years) of individual with MM		
0–9	–	6
10–19	1	8
20–29	2	0
30–39	1	0
40–49	7	0
50–59	5	1
60–69	2	0
MM diagnosis		
Clinical and biochemical evidence	9	3
Confirmed genetic etiology	9	12
Genetic Etiologies		
nDNA		
*ATP5MK*	0	1
*DLD*	1	0
*FBXL4*	0	2
*HSD17B10*	0	1
*NUBPL*	0	1
*OPA1*	0	1
*POLG*	1	0
*RARB*	0	1
*SUCLG1*	0	1
*SURF1*	0	1
*TWNK*	1	0
mtDNA		
SLSMD	1	0
*MT-ATP6* (m.9026G>A)	1	0
*MT-ND3* (m.10191T>C)	0	2
*MT-ND5* (m.13513G>A)	0	1
*MT-TL1* (m.3243A>G)	3	0
*MT-TL1* (m.3288A>G)	1	0

Subjects with symptoms consistent with MM were enrolled in an IRB-approved research study. This cohort includes subjects with a definitive genetic diagnosis as confirmed by the presence of pathogenic variant(s) in nuclear or mitochondrial DNA, and those highly suspected to have MM based on clinical and biochemical evidence without definitive genetic etiology confirmed to date.

MM, Mitochondrial Myopathy; mtDNA, mitochondrial DNA; nDNA, nuclear DNA; SLSMD, single large-scale mitochondrial DNA deletion.

### Description of themes identified

The codebook included specific medical, psychosocial, and subjective themes. In total, 8 themes with 41 sub-themes were highlighted by subjects (Table 2, Supplemental File 2). Inter-rater reliability of coded interview transcripts demonstrated an average agreement of 96.98%, with an overall unweighted kappa score of 0.55. Across the semi-structured interview, themes of medical and psychosocial aspects of MM and related consequences arose. Eight discrete groups of themes were identified that included: (i) ADLs, (ii) “other organ system involvement,” (iii) “other medical themes,” (iv) “adapting to life with MM,” (v) “circumstances,” (vi) “independence and relationships,” (vii) “severity,” and (viii) “other psychosocial and subjective” themes and encompassed a total of 41 sub-themes.

**Table 2.** Themes and sub-themes identified in interviews.

Specific medical themes				
Activities of daily living		Other organ system involvement		Other
Bathing		Cognition		Falls
Chores		GI		
Dressing		Hearing		
Driving		Mood		
Eating		Outward appearance		
Hobby		Pain		
School		Ptosis		
Shopping		Reproductive		
Sleep		Respiratory		
Socializing		Vision		
Stairs		Voice		
Therapies				
Walking				
Work				
Psychosocial and subjective themes				
Adapting to life with MM	Circumstances	Independence and relationships	Severity	Other
Adapting	Current ability	Communication	Minor	Worry
Need to take a break	Doing what I want	Doctors	Severe	
Planning ahead	What I used to do	Family		
Pushing past my limits		Comparison to others		
		Dependence		

Specific medical, psychosocial, and subjective themes emerged throughout the course of the interviews. In total, 8 themes with 41 sub-themes were mentioned across the study cohort. Sub-themes were grouped based on patterns identified among the themes.

GI, gastrointestinal involvement; MM, Mitochondrial Myopathy.

#### Specific medical themes

a. ADLs: The most common medical theme was ADLs (Supplemental Figure 1(A)). This encompassed 14 sub-themes, including walking (mentioned by 27/33 subjects at any point in the interview), hobbies (25/33), eating (21/33), chores (20/33), and socializing (20/33) being mentioned the most common, while driving (9/33) and therapies (6/33) were mentioned less often. We then ranked the ADLs by increasing order of difficulty, as informed by the ratio of work to resting metabolic rate for each ADL, the metabolic equivalent of task (METs),^40,41^ and clinical expertise (J.F., Z.Z.-C.; Supplemental Table 2). We observed a significant increasing trend in ADLs being mentioned in discussion (“subject counts”) as the task difficulty increased from easiest to hardest (Cochran-Armitage trend test, *p* < 0.05, Supplemental Figure 1(A)).
b. Other organ system involvement: “Other organ system involvement” encompassed 11 sub-themes with pain (mentioned by 23/33 subjects at any point in the interview), GI involvement (19/33), mood (18/33), cognition (12/33), and outward appearance (10/33) arising in multiple discussions (Supplemental Figure 1(B)). Hearing (5/33), ptosis (5/33), and reproductive concerns (1/33) were mentioned the least. Given the inability to rank sub-themes in this group for the Cochran-Armitage trend test, statistical analysis was not performed.
c. Other medical themes: More than 75% of subjects (25/33) discussed prior falls or concern for falls during the interview (Supplemental Figure 1(C)). Of note, 17/25 subjects with falls reported imbalance (Figure 6(C)).

#### Psychosocial and subjective themes

a. Adapting to life with MM: Themes of adapting to having MM symptoms arose, encompassing four subthemes with “need to take a break” most commonly mentioned (18/33 subjects at any point in the interview, e.g., one subject noted, “*. . . if we are writing a big paragraph I have to stop and take a break after a few sentences.*”) followed by “adapting” (12/33; e.g., one subject noted, “*When you were talking about climbing into a car, he would need to kneel down then climb in. We sold that car and got a lower car with buttons that open and close the doors because we felt that it wasn’t good for his self-esteem, even though he could get into the different car*.”) and “planning ahead” (12/33; e.g., one subject noted, “*I need to strategize my day, plan for things that I might not be able to get done. I can’t make calls when I’m jumbling my words. People think I’m drunk or having a stroke.*”; Supplemental Figure 1(D)).
b. Circumstances: Themes of circumstances were discussed, encompassing three sub-themes including when subjects referenced their “current abilities” (30/33 at any point in the interview), “doing what I want” (24/33; e.g., one subject noted, “*Sometimes I just feel sluggish or blah, I just don’t feel like moving or doing anything which is unusual for me. I don’t like just sitting on the couch doing nothing but sometimes I have to*”) and reflecting on “what I used to do” (13/33; e.g., one subject noted, “*I can’t play singles, I get too tired, but I can do doubles. I don’t play tennis as much as I used to anymore.*”; Supplemental Figure 1(E)).
c. Independence and relationships: Themes of independence and relationships were reported, encompassing five sub-themes including “dependence” on external entities such as people and/or assistive devices or services (23/33 subjects at any point in the interview; e.g., one subject noted, “*I’m more dependent on grocery delivery services instead of going to the store to go shopping. I really just don’t go shopping for anything anymore.*”), thoughts on how they see themselves or appear relative to others, or “comparison to others,” (17/33; e.g., one subject noted, “*I worry that someone might think I am drunk walking down the street.*”), “communication” (16/33; e.g., one subject noted “*His slow pace affects everything. His eating, his conversation, his talking, everything is slower. His brain takes longer time to process.*”), and “family” (13/33; e.g., one subject noted, “*If I push, push, push, and then I will crash. I can’t even get out of bed. I feel like I will miss out on family time.*”). Less commonly mentioned were doctors (7/33; Supplemental Figure 1(F)). Statistical analysis was not performed (Supplemental Figure 1(C)–(F)) due to low numbers.
d. Severity: Themes of severity encompassed two sub-themes. Approximately 50% reported some of their MM symptoms to be severe (17/33 subjects at any point in the interview; e.g., one subject noted, “*Exercise intolerance limits me every day from what I can do. Housework, yardwork, exercise to stay in shape—everyday it impacts my life. I can’t do things I want to do.*”*)* and 27% used terms indicating a less impactful effect of their MM symptoms (9/33, e.g., one subject noted, “*[my symptoms of neuropathy] don’t impact my daily life as in limiting activities but it is just another annoyance on the list*,” Supplemental Figure 1(C)).
e. Worry: “Worry” was mentioned by more than 50% of subjects (18/33), which was assigned to its own “other psychosocial and subjective themes” category (Supplemental Figure 1(C)).

### Interaction of themes

Some subject responses mentioned two or more themes concurrently, the most common being a medical theme (ADL or “other organ system involvement”) and its consequences or psychosocial relevance. These results were expressed as “frequency of theme co-occurrences” (Supplemental Figures 2 and 3, Table 3).

**Table 3.** Most common co-occurring themes in relation to impacted ADLs.

ADL	Most common co-occurring theme
Walking (*n* = 27)	
Adapting themes	Need to take a break (5/27, 19%)
Independence and relationship themes	Dependence (10/27, 37%)
Hobby (*n* = 25)	
Adapting themes	Need to take a break (5/25, 20%)
Independence and relationship themes	Comparison to others (5/25, 20%)
Eating (*n* = 21)	
Adapting themes	Adapting (5/21, 24%)
Independence and relationship themes	Communication (5/21, 24%)
Socializing (*n* = 20)	
Adapting themes	Planning ahead (6/20, 30%)
Independence and relationship themes	Communication (8/20, 40%)
Chores (*n* = 20)	
Adapting themes	Need to take a break (4/20, 20%), planning ahead (4/20, 20%)
Independence and relationship themes	Dependence (5/20, 25%)

Subjects often discussed co-occurring themes, and not themes in isolation.

ADL, activities of daily living.

#### Activities of daily living

ADLs were discussed in association with themes of “adapting to life with MM” (Supplemental Figure 2(A)), “circumstances” (Supplemental Figure 2(B)), “independence and relationships” (Supplemental Figure 2(C)), and severity (Supplemental Figure 2(D)). “Adapting” specifically was mentioned in relation to eating frequently (of the 21 times that eating was mentioned by any subject throughout the course of the interviews, it was mentioned in relation to “adapting” five times, 5/21, 24% (Supplemental Figure 2(A), purple bars), while walking was mentioned four times in relation to “adapting” (4/27, 14.8%). By comparison, driving (1/9, 11%), work (1/11, 9%), shopping (1/11, 9%), stairs (1/12, 8%), sleep (1/18, 6%), and hobbies (1/25, 4%) were rarely mentioned. Themes of “need to take a break” arose often on discussions of sleep (6/18, 33%), hobbies (5/25, 20%), and walking (5/27, 19%; Supplemental Figure 2(A), blue bars). Meanwhile, “planning ahead” was regularly discussed in the context of socializing (6/20, 30%; Supplemental Figure 2(A), green bars), however was seldom mentioned in relation to school (1/15, 7%) and sleep (1/18, 6%), and was never mentioned in response to bathing, dressing, and therapies. Subjects routinely mentioned hobbies (4/25, 16%), work (4/11, 36%), and walking (6/27, 22%) when considering “what I used to do” (Supplemental Figure 2(B), green bars). Hobbies (13/25, 52%), walking (11/27, 41%), chores (9/20, 45%), and socializing (8/20, 40%) were commonly associated with being able to do what they desired to do, or “doing what I want” (Supplemental Figure 2(B), blue bars). Pairwise comparisons by Fisher’s exact test between co-occurring themes (ADL themes co-occurring with “adapting to life with MM” themes, Supplemental Figure 2(A); ADL themes co-occurring with themes related to “circumstances,” Supplemental Figure 2(B)) did not reach statistical significance (all *p* > 0.05), likely due to low numbers.

Under the umbrella of “independence and relationships” (Supplemental Figure 2(C)), “communication” was mostly mentioned alongside eating (5/21, 24%) and socializing (8/20, 40%, purple bars). Subjects most often described themselves in “comparison to others” with respect to hobbies (5/25, 20%, Supplemental Figure 2(C), orange bars); for example, one subject noted “. . . *I have less motivation to go to the gym. Almost knowing that when I get there, I’m not going to last very long deters me, and there’s also some embarrassment since I can’t go as long as other people who are there at the gym.*”) or socializing ((5/20; 25%, Supplemental Figure 2(C)); for example, one subject noted “*His fatigue is generally characterized by slowing down, he cannot keep pace. For example, if he’s playing outside with other kids, he can’t keep up with them . . . he comes in much sooner*”). Pairwise comparisons by Fisher’s exact test between co-occurring themes (ADL themes co-occurring with “independence and relationship” themes, Supplemental Figure 2(C)) revealed a significantly higher mention of socializing when compared to walking on discussions related to communication (*p* < 0.05). All other pairwise comparisons were not significant.

Walking (7/27, 26%), eating (5/21, 24%), and chores (4/20, 20%) were commonly referred to as “severely” impacted ADLs (Supplemental Figure 2(D), blue bars). Dressing (1/12, 8%), driving (1/9, 11%), sleep (1/18, 6%), stairs (1/12, 8%), and therapy (1/6, 17%) were less frequently referred to as “severe,” and bathing and socializing were never described in severe terms. Pairwise comparisons by Fisher’s exact test between co-occurring themes (ADL themes co-occurring with themes centered on severity; Supplemental Figure 2(D)) did not reveal any significant differences (all *p* > 0.05).

##### Other organ system involvement

Themes of “adapting to life with MM” also arose in relation to “other organ system involvement.” “Pushing past my limits” was routinely mentioned in association with mood (of the 18 times mood was mentioned by any subject throughout the interviews, it was mentioned in relation to “pushing past my limits” four times (4/18, 22%); e.g., one subject noted, “*I try really hard to exercise at night, like taking a gym class with a friend, but I just can’t push as hard as they can sometimes. I notice they are pushing harder than me, I can’t go as long, and I get really sweaty. Sometimes it is a true bummer, I try to keep going but I just don’t know sometimes if I should put my energy into that*.” Supplemental Figure 3(A), *orange bar*).

Themes of “circumstances” similarly arose. Subjects regularly referenced their “current abilities” or “current status” (Supplemental Figure 3(B), purple bars) on discussing their GI involvement (8/19, 42%), cognition (7/12, 58%), pain (5/23, 22%), and mood (4/18, 22%). By comparison, past abilities (“what I used to do”) was alluded to on more than one occasion in relation to cognition (2/12, 17%; e.g., one subject noted, “*I used to love reading and now I can’t sit down and focus on reading a book*”) and vision (2/9, 22%; e.g., one subject noted, “*I also can’t drive anymore but I made that choice on my own because of the vision issues*,” Supplemental Figure 3(B), green bars).

Themes centered on “independence and relationships” arose in relation to “other organ system involvement” (Supplemental Figure 3(C)). “Communication” was brought up alongside cognition (6/12, 50%; e.g., one subject noted, “[MM] *slows me down cognitively. I can’t follow a long, in-depth conversation.*”), hearing (4/5, 80%; e.g., one subject noted, “*I would like a significant change—I would be able to speak on the phone better and watch TV without closed captioning and be able to speak with my friends and family better. Another issue is obviously safety. I take my hearing aids out at night and at that point I can’t hear anything.*”), vision (3/9, 33%; e.g., one subject noted, “*I can’t see so I am sending personal messages to wrong people. It is very embarrassing.*”), and voice (7/8, 88%; e.g., one subject noted, “*If I speak very loudly, projecting my voice is very difficult and I run out of breath similar to if I walk too far.*”; Supplemental Figure 3(C), purple bars). Pairwise comparisons by Fisher’s exact test revealed that voice was discussed significantly more than ptosis (*p* < 0.05), Supplemental Figure 3(C), purple bars).

Concepts centered on “comparison to others” also arose in conjunction with mood (6/18, 33%; e.g., one subject noted, “*It’s hard enough not to get depressed. I’m lucky—my brother died, my cousin died. They both had mito disease. I’m fortunate. If you look at me, you can’t tell I have this*”), outward appearance (2/10, 20%; e.g., one subject noted, “*[An improvement would be] being able to walk normally without other people noticing I have a balance problem.*”), and cognition (2/12, 17%; e.g., one parent noted, “*You can tell she has to think hard about a more complicated physical task—it is simple for us, just a two-action task. It’s really the coordination of actions.*”, Supplemental Figure 3(C), *orange bars*). Pairwise comparisons by Fisher’s exact test revealed that mood was mentioned significantly more frequently than “GI” symptoms (*p* < 0.05), Supplemental Figure 3(C), orange bars).

A description of “severe” was often used on describing pain (8/23, 35%; e.g., one subject noted, “*It’s terrible, I want to have a job, but I can’t because my legs hurt so bad. It is something you can’t push through*”), “GI” involvement (4/19, 21%; e.g., one parent noted, “*She can’t eat or she can’t tolerate her feeds. There have been times where she hasn’t been able to grow as a result of not being fed as much as she needs. Her ‘GI’ issues are definitely the biggest symptom because it causes her incredible pain. It affects all our lives.*”), and vision (3/9, 33%; e.g., one subject noted, “*Reading anything and using the computer [is affected]. When I was working, I was working in IT, so it was really affecting my work.*”), as compared to only one mention each on describing voice (1/8), cognition (1/12), and mood (1/18), and was not mentioned in relation to hearing, outward appearance, ptosis, reproductive, or respiratory involvement (Supplemental Figure 3(D), blue bars).

Pairwise comparisons by Fisher’s exact test for co-occurrence of themes centered on “other organ system involvement,” themes of “circumstance” and “severity” (Supplemental Figure 3(A), (B), and (D)) did not reveal any significant differences (all *p* > 0.05).

### Definition of MM across all symptoms, impact of MM, and what is considered a slight improvement

#### MM characterization

Subject responses to being asked to *define* their main MM symptoms, discuss the *impact* of MM, and explain what a slight *improvement* would look like were variable across the MM domains of muscle weakness, muscle fatigue, exercise intolerance, imbalance, and peripheral neuropathy (Figure 2). Results are presented as the cumulative number of times (“frequency”) a theme was mentioned throughout the interview and not as subject counts, since subjects with symptoms in more than one MM domain may have mentioned a particular theme in one or more domains. For example, a subject with impaired mobility may have mentioned walking on discussing both exercise intolerance as well as muscle fatigue. Thus, themes that arose in discussion were not exclusive to specific MM domains.

#### Definition of MM across all symptoms

When asked to *define* their main MM symptoms, walking, which was mentioned 94 times across the study cohort interviews was discussed the most often across all ADLs (19 times of 94 mentions, 20%; e.g., a subject with exercise intolerance noted, “*When I walk long distances, if I walk five minutes, my legs start burning and I have to sit down*,”), followed by sleep (13/37 times, 35%, e.g., a subject with muscle fatigue noted, “*Physically, mentally, and emotionally the fatigue is overwhelming . . . waking up feeling like you need to go to bed, or like you ran a marathon in your sleep is just exhausting*.”), and hobbies (10/54 times, 19%, e.g., a subject with exercise intolerance noted, “*I don’t have the energy to play tennis like I used to*,”) as compared to driving (1/14, 7%) and other ADLs that were mentioned less than seven times in response to being asked to *define* MM symptoms (Figure 2(a), purple bars). Of note, there were additional open-ended questions that were asked outside the scope of *definition, impact*, and *improvement*, therefore the total numbers displayed across *definition, impact,* and *improvement* in all figures (e.g., Figure 2(a)) do not constitute the total frequencies. Pairwise comparisons by Fisher’s exact test between ADLs in response to *definition* of MM questions did not reach significance (*p* ⩾ 0.05, Figure 2(a), purple bars).

Themes related to “other organ system involvement” also arose, with “pain” significantly mentioned the most (26/67 times, 39%) as compared to “GI” symptoms (2/54 times, 4%), *p* < 0.001 (Figure 2(b)) on discussing the *definition* of MM symptoms. “Respiratory [involvement]” was also noted significantly more in the *definition* of MM symptoms as compared to “GI” symptoms, *p* < 0.001 (Figure 2(b), purple bar). “Mood” and “outward appearance” were also mentioned multiple times. Additional pairwise comparisons by Fisher’s exact test between “other organ system involvement” themes in response to the *definition* of MM questions were not significant (*p* ⩾ 0.05, Figure 2(b), purple bars).

“Falls” was mentioned 20 times in relation to the *definition* of symptoms, while “severe” and “mild” were mentioned at the same frequency of 5 times (Figure 2(c), purple bars). “Worry” was only mentioned four times in relation to the *definition* of MM symptoms (e.g., a subject with neuropathy noted, “*If I drop something on my feet, I can’t feel it. I have a lot of bruising on my toes because I just don’t feel it. It feels dangerous*,”). Pairwise comparisons were not performed due to low numbers (Figure 2(c)).

Themes related to “adapting to life with MM” were noted on being asked to *define* symptoms (Figure 2(d), purple bars). “Needing to take a break” was regularly mentioned at 11 times (of 33 mentions across the cohort interviews, 33%) and “pushing past my limits” at seven times (of 18 mentions, 39%) but was not significantly higher (*p* > 0.05) as compared to “planning ahead” that was only mentioned once (1/13, 8%). For example, in terms of “need to take a break,” one subject with exercise intolerance stated “*[The main symptoms I experience are] muscle fatigue, winded easily, shortness of breath, feeling of weakness. It doesn’t take long for me to have to stop and take a breath*;” while a subject with muscle fatigue defined MM as, “*I went to [a grocery store] yesterday and [after] the walk from the handicap parking space to the aisle, I had to stop. It is as if I need to recover. . .*”*).* Themes that centered specifically on “adapting” were consistently and recurrently brought up upon asking subjects to *define* their MM symptoms (5/14 times that “adapting” was mentioned, 36%, Figure 2(d)). For example, in response to being asked to *define* muscle fatigue, one subject noted a specific theme of “adapting,” “*We . . . have a dictation system set up for him with speech to text.*” Pairwise comparisons by Fisher’s exact test between themes centered on “adapting to life with MM” in response to *definition* of MM questions were not significant (*p* ⩾ 0.05, Figure 2(d), purple bars).

Themes that centered on “circumstances” were also noted on *defining* MM (Figure 2(e)). Pairwise comparisons by Fisher’s exact test were not significant (*p* ⩾ 0.05, Figure 2(e), purple bars).

In terms of themes centered on “independence and relationships,” reliance on external entities, or “dependence,” was noted in the *definition* of MM symptoms seven times by subjects (e.g., a subject with exercise intolerance noted, “*I just feel extremely weak if I try to walk too long or travel. I have to use a cane or sit and lean on something because my body just doesn’t feel strong enough to keep going.*”; Figure 2(f), purple bars). Pairwise comparisons were not performed due to low numbers.

#### The overall impact of MM symptoms

When asked about the *impact* of MM, ADLs were discussed more broadly, with every ADL except shopping and therapies being mentioned 10 times or more at any point in the interview across the study cohort (Figure 2(a), blue bars). Pairwise comparisons by Fisher’s exact test between ADL themes on discussions of MM *impact* were not significant (*p* ⩾ 0.05, Figure 2(a), blue bars).

In “other organ system involvement,” “pain” was a significantly more frequent theme cited in response to *impact* (11/67 times, 16%, e.g. one parent noted in response to the question regarding impact of imbalance, *“He hurts himself a lot when he falls.”*) followed by “outward appearance” (7/15, 47%, e.g., one subject in response to the question regarding *impact* of muscle weakness, “*I also have ptosis, and I’m not sure if that is considered muscle weakness. It bothers me a lot in how it affects my appearance, but it also obstructs my vision, which is just an annoyance.*”). “Vision” (5/23, 22%), “mood” (5/33, 15%), and “GI symptoms” (5/54, 9%) were also mentioned. Indeed, pairwise comparison by Fisher’s exact test demonstrated that “pain” was mentioned significantly more than “outward appearance” on discussing the *impact* of MM. No other themes arose significantly more frequently as compared to one mention of the reproductive system (1/2, 50%, *p* > 0.05, Figure 2(b), blue bars).

“Falls” were mentioned 16 times, “worry” and “severe” were described 11 times, and “mild” noted 9 times, in response to *impact* (Figure 2(c), blue bars). In terms of “worry,” a subject with exercise intolerance noted, “*I have always been active and can’t be as active as I want. My kids are young now and I can still beat them in a foot race and I expect to be doing that for a long time but don’t know how long I can expect to do that.*” and a subject with muscle fatigue noted, “*I get kind of foggy when I’m fatigued, so I don’t know if I can think everything through quickly if I need to. I don’t want to be driving and then run a stop sign or anything unsafe like that*”. Pairwise comparisons were not performed due to low numbers (Figure 2(c), blue bars).

Themes of “adapting to life with MM” occurred at similar frequencies, with “adapting” referred to nine times, and “needing to take a break,” “planning ahead,” and “pushing past my limits” each mentioned seven times (Figure 2(d), blue bars). For example, in terms of “planning ahead,” a subject with muscle fatigue noted in response to *impact*, “*I also need to plan for when I will be able to drive. I have to think, is this really worth it? Will I be able to get home?*” Pairwise comparisons by Fisher’s exact test between themes centered on “adapting to life with MM” in response to MM *impact* questions were not significant (*p* ⩾ 0.05, Figure 2(d), blue bars).

Themes of “circumstances” were also frequently mentioned, “current ability,” 40 times, “doing what I want,” 29 times, and “what I used to do,” 9 times (Figure 2(e)). One subject, when asked about the *impact* of fatigue, described the impact on “doing what they want,” such as “*It affects my life because I have to turn things down, like walking across the street to lunch. I say I can’t go. It affects socializing and getting things done like errands.*” Similarly, subjects often compared current abilities to past abilities, “what I used to do,” (e.g., a subject with exercise intolerance noted in response to *impact*, “*I used to ski. I used to hike. I don’t do those things anymore*,”and a subject with fatigue noted, “*I can no longer work. When I was working, I would work from home and need to take two or three naps throughout the day to get through it. That really impacted my life, the fatigue does not allow me to hold down a full-time job*”). Pairwise comparisons by Fisher’s exact test between themes centered on “circumstances” in response to MM *impact* questions were not significant (*p* ⩾ 0.05, Figure 2(e), blue bars).

Across the themes of “independence and relationships,” “dependence” was regularly discussed at 21 times (e.g., a parent of an individual with muscle weakness noted, “*His arms are weak right now that I have to feed him. He will take one or two bites and then become too weak*,” and a subject with fatigue noted, “*Washing dishes, housekeeping. I have a personal caregiver that has to help with these things. I have had a housekeeper/caregiver for 10-11 years*,” whereas another subject with muscle fatigue noted, “*Recently I have had to move back in with my family also because I just can’t get it all done on my own anymore*,” and a parent of an individual with imbalance stated, “*He can’t walk, he can’t stand, he can’t go upstairs. He hasn’t been upstairs in probably 15 years, if not longer. He’s always in the wheelchair, that’s his life*,” while another individual with imbalance noted, “*[I am impacted] walking in front of strangers at all. I just don’t do that. I’ll hold my daughter’s or my husband’s hand so that I don’t veer off left.*”). “Communication,” “family,” and “comparison to others” were each mentioned eight to nine times (e.g., a subject with exercise intolerance noted the following about family, “*When our kids were little, I would park and walk to pick up the kids, but now that they’re older I’m always in the pick-up line. I would love to park and be able to walk to get them*;” and a subject with muscle fatigue noted, “*It’s tough for me to endure exercise—mowing the lawn, going to the park with the kids, doing some work around the house. I have to pace myself*”). “Doctors” was mentioned three times (Figure 2(f), blue bar). Pairwise comparisons were not performed due to low numbers (Figure 2(f), blue bars).

#### What would be considered a meaningful slight improvement

When asked what a meaningful slight *improvement* would be, responses were focused on specific ADLs, including walking (26/94 times, 28%; Figure 2(a), green bars, e.g., a subject with fatigue noted, “*Anything that could allow me to walk longer would mean a lot to me. I can only walk a short distance. Fifty more feet would be an improvement. My independence and freedom are taken away because I can’t walk far*,”), hobbies (17/54 times, 31% e.g., a subject with muscle fatigue noted, “[*A slight improvement would be*] *if I were to go to the rock gym to climb. I used to spend three hours there and even if I had that time, between work and family obligations, I wouldn’t be able to spend more than a half hour there*,”), chores (13/49 times, 27%, e.g., a subject with muscle weakness noted, “[*A slight improvement would be*] *if I could do more sorting . . . or folding laundry. It would be better to have longer endurance with these types of tasks. I always have to stop—my arms feel weak*,”), eating (11/57 times, 19%; e.g., a parent of a child with fatigue noted, “[*A slight improvement would be*] *being able to eat by mouth and taste and enjoy food.*”), and socializing (10/44 times, 23%; e.g., a parent of a subject with exercise intolerance noted, “[*A slight improvement would be*] *being able to be more interactive and participate in conversations. [He] gets too tired to sit and talk at the dinner table*”). Also noted was dressing (6/22 times, 27%; e.g., a parent of a subject with muscle weakness noted, “[*A slight improvement would be*] *he would be able to style his hair—he’s a teenage boy, he wants to style it with mousse, and I would love to see him be able to zip his own coat*,”), school (6/40 times, 15%; e.g., a parent of a child with exercise intolerance noted, “[*A slight improvement would be for him to] complete his routine throughout the day. If we improve his exercise intolerance we could make it to a 3pm class in addition to the other daily activities*,”), bathing (5/24, 21%; e.g., a subject with muscle fatigue noted, “[*A slight improvement would be*] *getting ready in the morning. The process of getting up, showering, brushing teeth, combing hair, making breakfast—that is all really fatiguing. That would be something I would recognize every day, seeing or noticing an improvement*,”), and sleep (5/37 times, 14%; e.g., a subject with exercise intolerance noted, “[*A slight improvement would be*] *longer duration with activity without as much fatigue—getting through a five-hour work day without dozing off*,”). Not surprisingly, again due to low numbers, no significant difference was observed (*p* > 0.05) when each of the above mentioned ADLs was compared to only one mention of driving (1/14, 7%, Figure 2(a), green bars).

Subjects also elaborated on themes related to “other organ system involvement” (Figure 2(b), green bars), such as “pain” (13/67, 19%) and “cognition” (5/26, 19%), which were not significantly different (*p* > 0.05) from “vision” (1/23, 4%) that was less frequently discussed.

“Falls” were noted 20 times (20/67, 30%) in relation to desired i*mprovement* and “worry” was noted three times (3/32, 9%; e.g., a subject with exercise intolerance noted, “[*A slight improvement would be] being able to feel like I can do an exercise routine, to have a comfortable idea of where my threshold is so I don’t have to worry about messing myself up*”), Figure 2(c), green bars). Pairwise comparisons were not performed due to low numbers (Figure 2(c), green bars).

“Adapting” was not discussed in relation to *improvement* but was deliberated in the context of “need to take a break” 11 times (11/33, 33%; Figure 2(d), green bars). A subject with muscle fatigue described, “*[A slight improvement would be] just pushing the fatigue back a bit. Being able to go for a little longer before needing to rest. I currently feel fatigued maybe three hours after I wake up in the morning. A five hour stretch of being able to go would be great;*” while a subject with exercise intolerance noted, “*[A slight improvement would be] being able to use a mouse or computer a little longer without having to rest my hand or stretch*,”). Pairwise comparisons were performed between themes centered on “adapting to life with MM” in response to questions on *improvement* and were not significant (*p* ⩾ 0.05, Figure 2(d), green bars).

Themes of “doing what I want” arose 21 times (21/79, 27%) in relation to desired *improvement* compared to just two occurrences of “what I used to do” (2/21, 10%; Figure 2(e), green bars). Pairwise comparisons by Fisher’s exact test were performed and none were significant (*p* ⩾ 0.05, Figure 2(e), green bars).

“Dependence” was mentioned 13 times (13/50, 26%) when asked what a slight *improvement* would be (e.g., a subject with fatigue noted, “*[A slight improvement would be] being able to get my own food and not have someone need to bring it to me*,” while a subject with exercise noted, “*[A slight improvement would be to] take a walk and then take the dogs for another walk, not having to depend on someone else to do it*,” and a parent of a child with muscle weakness noted, “[*A slight improvement would be] being able to walk with a walker and have more endurance with the walker. He can use the walker now, but he can’t go very far*”). “Family” was highlighted in response to being asked about *improvement* four times (4/23, 17%; e.g., a subject with fatigue noted, “*[A slight improvement would be] having a little more stamina to be able to do more in a day . . . if I know my family is going to dinner on Saturday night I will lay low all day Saturday just to make it to dinner that night*,” Figure 2(f), green bars). Pairwise comparisons were not performed due to low numbers (Figure 2(f), green bars).

### Characterization of individual MM domains

#### Within MM domain characterization

Subjects were asked whether they experienced symptoms in each of the five MM domains using close-ended questions (Supplemental Figure 4). The most frequently reported symptom was muscle fatigue (present in 31/33, 94%), followed by exercise intolerance (29/33, 88%), muscle weakness (27/30, 90%), imbalance (19/33, 58%), and peripheral neuropathy (15/33, 45%). Results of thematic analysis in each domain are presented as subject counts, representing the number of subjects who stated that they experienced symptoms in a particular MM domain and discussed a given theme. Walking was the most commonly referenced ADL (Table 4).

**Table 4.** Most mentioned ADL themes across MM domains.

Domain	Most common ADL themes
Muscle weakness (*N* = 27)	
Impact	Dressing (7/27, 26%)
Improvement	Walking (7/27, 26%)
Exercise intolerance (*N* = 29)	
Impact	Walking (12/29, 41%)
Improvement	Walking (11/29, 38%)
Fatigue (*N* = 31)	
Impact	Chores, socializing, walking (each 10/31, 32%)
Improvement	Eating (6/31, 19%)
Imbalance (*N* = 19)	
Impact	Walking (11/19, 58%)
Improvement	Walking (5/19, 26%)
Neuropathy (*N* = 15)	
Impact	Driving (4/15, 27%)
Improvement	–

ADL themes recurred across MM domains. No recurrent themes were discussed in relation to desired improvement in peripheral neuropathy.

ADL, activities of daily living; MM, mitochondrial myopathy.

#### Muscle weakness

Across the study cohort, 3/33 subjects did not provide a response on whether they had muscle weakness. Meanwhile, another 3/33 subjects explicitly stated they did not have muscle weakness. Therefore, 27/30 (90%) subjects who were asked if they had muscle weakness responded affirmatively and responded to the open-ended questions. However, 3/27 subjects with muscle weakness provided incomplete responses to the muscle weakness-specific closed-ended questions. All 27 subjects responded to where they were weakest. Only one subject did not answer any of the other closed-ended questions. Two subjects provided incomplete responses, including missing responses for climbing into car, getting dressed, standing in the shower, standing while cooking, standing while waiting for a bus, standing without support, and walking.

Results of close-ended questions in subjects with muscle weakness revealed that weakness was noted while standing waiting for a bus (12/24, 50%), cooking (8/24, 33%), showering (8/24, 33%); and during self-hygiene activities that included grooming hair (11/26; 42%), eating (8/26; 31%), and dressing (6/24, 25%). We then ranked the listed tasks by increasing order of difficulty, as informed by METs values^40,41^ and MM clinical expertise (J.F., Z.Z.-C.; Supplemental Table 3). We observed a significant increasing trend (Cochran-Armitage trend test *p* < 0.05) in the number of subjects who noted difficulty with increasingly harder tasks (Figure 3(a)). Additionally, 10/27 (37%) reported their arms as being weakest, 9/27 (33%) their legs, and 8/27 (30%) reported weakness in both arms and legs. Further, 19/24 (79%) subjects were ambulatory and 18/24 (75%) were able to stand without support (Figure 3(a)).

In the open-ended interview questions, subjects with muscle weakness (*n* = 27) described *impact* across multiple ADLs including dressing (7/27, 26%), chores (6/27, 22%), eating (5/27, 19%), and walking (5/27, 19%; Figure 3(b), blue bars). Additionally, individuals repeatedly discussed *impact* with “other organ system involvement” (Figure 3(c)), such as their “outward appearance” (5/27, 19%) and “vision” (3/27, 11%). Least mentioned were “GI” symptoms (1/27, 4%) and “mood” (1/27, 4%, blue bars). All pairwise comparisons between themes were not significant, *p* ⩾ 0.05 (Figure 3(b), blue bars).

“Falls” and “severe” were noted five times (each 5/27, 19%) as they relate to the *impact* of having muscle weakness (Figure 3(d), blue bars). “Adapting” was frequently noted (5/27, 19%, Figure 3(e), blue bars) and the *impact* of muscle weakness was also discussed in the context of “current ability” (12/27, 44%) as well as “doing what I want” (4/27, 15%) and “what I used to do” (2/27, 7%; Figure 3(f), blue bars). Lastly, muscle weakness *impacted* “dependence” on others and technology (Figure 3(g), blue bars), and this was a desired area of *improvement* (Figure 3(g), green bars). Pairwise comparisons by Fisher’s exact test were not performed due to low numbers (Figure 3(d)–(g)).

#### Exercise intolerance

Subjects with exercise intolerance (*n* = 29) most commonly *defined* this domain by referring to its *impact* on walking (9/29, 31%, *p* < 0.05) as compared to the least commonly mentioned themes of bathing, school, socializing, and stairs (each 1/29, 3%, Figure 4(a), purple bars). Chores, dressing, driving, eating, shopping, sleep, and work were not mentioned at all in relation to the *definition* of exercise intolerance. Fisher’s exact test comparing hobbies (6/29 times) to bathing, school, socializing, stairs (each 1/29) was not significant, *p* = 0.1. When asked to describe the *impact* of exercise intolerance, walking (12/29, 41%) and hobbies (10/29, 34%) were each mentioned significantly more (*p* < 0.05) than driving, therapies, and work (each 2/29, 7%, blue bars). Similarly, on being asked what a slight *improvement* would be, walking (11/29, 38%) was mentioned significantly more frequently (*p* < 0.001) as compared to school, shopping, sleep, therapies, and work (each 1/29, 3%; Figure 4(a), green bars). However, Fisher’s exact test comparing hobbies (7/29 times) to school, shopping, sleep, therapies, and work (all 1/29) was not significant, *p* = 0.05. Similarly, Fisher’s exact test comparing socializing (5/29 times) to school, shopping, sleep, therapies, and work (each 1/29) was also not significant, *p* = 0.19 (Figure 4(a), green bars).

Notably, 13/29 (45%) subjects with exercise intolerance referenced “pain” significantly more in their *definition* of exercise intolerance compared to “respiratory involvement” (4/29, 14%), “mood” (3/29, 10%), and “outward appearance” (3/29, 10%), each *p* < 0.05 by Fisher’s exact test (Figure 4(b), purple bars). In terms of the *impact* of exercise intolerance, “pain” was a regularly discussed “other symptom” in relation to a meaningful slight *improvement* (5/29, 17%, Figure 4(b), green bar), while “cognition” and “respiratory” (each 1/29, 3%) were mentioned less often (Figure 4(b), green bars). Other common themes discussed in those with exercise intolerance included “worry” in relation to *impact* mentioned by 5/29 subjects (17%, Figure 4(c), blue bar), “needing to take a break” mentioned in relation to *definition* of exercise intolerance 5/29 (17%, Figure 4(d) purple bar), *impact* on doing desired things in 15/29 (52%, Figure 4(e), blue bar), and the *impact* on “dependence,” 5/29 (17%, Figure 4(f), blue bar). Statistical analysis was not performed to assess for significant differences in the occurrence of these themes due to low numbers.

#### Muscle fatigue

In contrast, subjects with muscle fatigue (*n* = 31) *defined* their symptom of fatigue in the context of sleep (12/31, 39%) significantly more often (*p* = 0.001) as compared to bathing, driving, therapies, walking, and work (each 1/31, 3%, Figure 5(a), purple bars), whereby the *impact* of muscle fatigue was noted more uniformly across ADLs including chores (10/31, 32%), eating (9/31, 29%), hobbies (6/31, 19%), school (8/31, 26%), sleep (8/31, 26%), socializing (10/31, 32%), walking (10/31, 32%), and work (8/31, 26%) Figure 5(a), blue bars). Subjects with muscle fatigue described eating (6/31, 19%), hobbies (4/31, 13%), school (4/31, 13%), sleep (4/31, 13%), and socializing (4/31, 13%) as areas of desired *improvement* (Figure 5(a), green bars).

“Cognition” was described by several subjects across all questions that assessed the *definition, impact*, and slight *improvement* of muscle fatigue (Figure 5(b)), as were “GI concerns,” “outward appearance,” and “voice” (Figure 5(b)). Pairwise comparisons were performed between themes centered on “other organ system involvement” and were not significant, *p* ⩾ 0.05 (Figure 5(b)).

“Worry” was mentioned in relation to *impact* in 3/31 (10%) subjects with muscle fatigue (Figure 5(c), blue bar). “Need to take a break” and “planning ahead” were mentioned across all questions of *definition, impact*, and slight *improvement* by multiple subjects (Figure 5(d)). “Doing what I want” and “comparison to past abilities” was also mentioned by at least one subject in response to every question centered on muscle fatigue (Figure 5(e)). “Communication” also commonly arose in the narratives of the *definition* of muscle fatigue and its *impact* (5/31, 16%, and 6/31, 19%, respectively), as were “family” and “dependence” which were discussed by multiple subjects in response to each question related to fatigue (Figure 5(f)). Pairwise comparisons were not performed due to low numbers (Figure 5(c)–(f)).

#### Imbalance

Subjects who reported imbalance (*n* = 19) elaborated on fewer ADLs in the *definition* and *impact* of imbalance, except for walking (11/19, 58%) and stairs (5/19, 26%) that were talked about on describing the *impact* of imbalance. Notably, walking (11/19, 58%) was mentioned significantly more (*p* = 0.001) as compared to driving, eating, school, shopping, and therapies (each 1/19, 5%, Figure 6(a), blue bars). Hobbies (3/19, 16%) and walking (5/19, 26%) were occasionally mentioned as areas of meaningful slight *improvement* (Figure 6(a), green bars).

Themes related to “other organ system involvement” were only mentioned by one subject in response to each question, if at all (Figure 6(b)). Pairwise comparisons were not performed due to low numbers (Figure 6(b)).

“Falls” were mentioned by multiple subjects in response to all questions of *definition, impact*, and slight *improvement* of imbalance (Figure 6(c)). Themes related to “adapting” were only mentioned by one subject, or none (Figure 6(d)). Themes of “current ability” arose in discussion in several subjects in response to *definition* and *impact*, while themes of “past abilities” arose less frequently (Figure 6(e)). “Dependence” was cited by multiple subjects (Figure 6(f)). Pairwise comparisons were not performed due to low numbers (Figure 6(c)–(f)).

#### Peripheral neuropathy

Subjects with peripheral neuropathy (*n* = 15) did not consistently define this domain in terms of ADLs. However, several subjects noted its *impact* on driving (4/15, 27%), school (2/13, 15%), and walking (3/15, 20%) (Figure 7(a), blue bars). Pairwise comparisons were performed between themes centered on ADLs using Fisher’s exact test and were not significant, *p* ⩾ 0.05 (Figure 7(a)).

“Pain” was routinely mentioned in “other organ system involvement,” noted by 10/15 (67%) in the *definition* of neuropathy, which was significantly more (*p* = 0.001) than “respiratory [involvement]” (1/15, 7%, Figure 7(b), purple bars). Meanwhile, 5/15 (33%) subjects mentioned pain in relation to *impact* (Figure 7(b), blue bar) and 6/15 (40%) in relation to desired *improvement* (Figure 7(b), green bar).

Themes related to “minor” severity were mentioned across all questions (Figure 7(c)). Themes related to “adapting” (Figure 7(d)), “current abilities” (Figure 7(e)), and “others” (Figure 7(f)) were rarely discussed, if at all. Pairwise comparisons were not performed due to low numbers (Figure 7(c)–(f)).

### Clinical trial motivation

#### Symptoms that motivated clinical trial participation

When asked about symptoms that would motivate participation in a clinical trial, all subjects who reported exercise intolerance (29/29, 100%), followed by muscle weakness (26/27, 96.3% of subjects with muscle weakness would participate in a trial), muscle fatigue (28/31, 90.3%), imbalance (16/19, 84.2%), and peripheral neuropathy (11/15, 73.3%; Figure 8(a)) reported willingness to participate. Further, ADL themes of walking (6/94, 6%), sleep (4/37, 11%), and chores (3/49, 6%) were commonly mentioned in response to being asked which symptoms should be addressed in a clinical trial (Figure 8(b)). Pairwise comparisons were performed between themes using Fisher’s exact test and were not significant, *p* ⩾ 0.05 (Figure 8(b)).

## Conclusion

We conducted this single-center, observational study seeking to characterize how MM patients feel about their symptoms in the key MM domains^27^ and to determine their functional ability through standardized structured and semi-structured interviews. To our knowledge, this is the first qualitative study to evaluate MM patient perspectives of the individual domains of muscle weakness, muscle fatigue, exercise intolerance, imbalance, and peripheral neuropathy, including its impact on ADLs.

Cohort interviews included open-ended questions to facilitate unbiased, unrestricted subject responses. No clarifying questions were asked if the subject did not elaborate their response, to maintain integrity of their narrative and avoid misinterpretation. Thus, responses in some subjects were shorter than others (e.g., one subject responded with only “weakness and tiredness” when asked about their exercise intolerance). This accounts for the variability in the frequency of themes mentioned across the cohort. All interviews were transcribed and qualitatively analyzed for common themes, followed by quantitation using a mixed-methods approach^38^ to quantify interview responses for comparison across and within key MM domains.

Subjects commonly *defined* their MM symptoms, described the *impact* of these symptoms, and what a slight *improvement* would be in the context of ADLs. A discussion of themes identified across the study cohort is included in Supplemental File 3. In addition, a description of the *definition, impact*, and desired slight *improvement* of the overall MM diagnosis is included in Supplemental File 4.

## MM characterization in each domain

The most frequently reported MM symptom was muscle fatigue (present in 31/33, 94%), followed by exercise intolerance (29/33, 88%), muscle weakness (27/30, 90%), imbalance (19/33, 58%), and peripheral neuropathy (15/33, 45%).

## Muscle weakness

Responses to the close-ended questions revealed that half of all subjects (12/24, 50%) with muscle weakness reported weakness on standing to wait for a bus. One-third of those subjects with muscle weakness reported weakness on standing to cook or on standing in the shower. In comparison, shorter “burst” activities, such as getting dressed and climbing into a car were only reported in 12%–16% of subjects with muscle weakness. This was also observed in the open-ended questions assessing *impact* and desired *improvement* in muscle weakness. Indeed, >20% of subjects with muscle weakness volunteered dressing and chores as being challenging. This is exemplified by subjects who described the *impact* of their muscle weakness: “*Shaving can be really difficult. I will only shave every 3 days*” and “*I’ve done laundry my whole life but now it exhausts me to lift wet clothes out of the washer. I used to lift a lot at once, and now I have to lift a piece or two at a time, but that repetitive movement then makes my arms tired, as well.*” Subjects repeatedly referenced these short-burst activities in relation to their muscle weakness. However, walking was the most frequently mentioned ADL in terms of how subjects envisioned a slight *improvement*, exemplified by one parent of an individual with MM who noted, “*[A slight improvement would be] being able to walk with a walker and have more endurance with the walker.*” This demonstrates the overarching desire of affected MM individuals to remain ambulatory.

“Outward appearance” was mentioned more frequently in those with muscle weakness than any other domain when asked about *impact*. This is exemplified by one subject who described how their weakness changed how they maintain their outward appearance, “*One of the things that has changed for me is that I don’t worry about showering every day or styling my hair. I need to put my energy into the household things like doing the dishes and taking care of the laundry*.” Indeed, muscle weakness not only affects level of functioning in daily activities but also self-perception.

Similar to “outward appearance,” “adapting” was broached more frequently in relation to the *impact* of muscle weakness as compared to any other domain. Subjects described how they adapted to having muscle weakness, “*Even putting in my contacts, I don’t have the strength and dexterity to put my contact lenses into my eye. I have to rest my arm on the counter and lower my head to my hand to put in a contact.*” This highlights the critical need in clinical studies and/or intervention trials to ask individuals with MM whether they were able to complete a daily activity with ease or difficulty and whether adaptions were required instead of a goal-oriented questionnaire that only asks whether they were able to achieve the task, as some MM individuals have adapted to their physical limitations.

## Exercise intolerance

Consistent with the overall study cohort, walking was the most commonly discussed theme in subjects with exercise intolerance as compared to bathing, school, socializing, and stairs, *p* < 0.05, Fisher’s exact test, Figure 4(a), followed by hobbies. Indeed, 9/29 (31%) of subjects with exercise intolerance reported difficulty walking and 6/29 (20.7%) reported restrictions of their hobbies. No subject mentioned chores, dressing, driving, eating, shopping, sleep, or work in their *definition* of exercise intolerance; and no more than two subjects brought up bathing, school, socializing, stairs, and therapies. However, walking and hobbies were emphasized on discussing the *impact* of exercise intolerance, as were chores, dressing, school, shopping, socializing, and stairs, demonstrating the broad impact of exercise intolerance in MM. Interestingly, walking, hobbies, and socializing were noted by several subjects on considering a desired *improvement*.

Themes centered on “other organ system involvement” arose less commonly in those with exercise intolerance with the notable exception of pain. Subjects consistently reported pain in the *definition* of their exercise intolerance (13/29, 45%) and a desired *improvement* (5/29, 17%).

Several subjects both *defined* exercise intolerance and described its *impact* by comparing themselves to other individuals. Similarly, several subjects described *impact* and desired *improvement* as reliance on external factors. “Doing what they wanted” was reported in *definition, impact*, and desired *improvement*. “Adapting” was less frequently discussed, in alignment with the strong motivation across subjects to participate in a clinical trial to treat exercise intolerance.

Subjects also brought up other themes when describing the *impact* of exercise intolerance, including 5/29 (17%) who noted an aspect of worry and 3/29 (10%) who noted “severe” impact. Exercise intolerance limited more than half of the study cohort from “doing things they wanted to do,” the highest observed frequency among all MM domains, exemplifying the pervasive and burdensome nature of exercise intolerance. Indeed, exercise intolerance may limit physical capability and mobility, which in turn affects mood and QOL.

## Muscle fatigue

Subjects with muscle fatigue (*n* = 31/33) described this symptom to be distinct from exercise intolerance, as the main descriptions of fatigue centered around sleep, the need for naps, and impaired ability to socialize. However, their *definitions* of muscle fatigue encompassed a broad range of ADLs except for dressing and stairs (Figure 5(a)). On describing the *impact* of muscle fatigue, this was the MM domain where social isolation and “dependence” where highlighted. This is consistent with results of a recent study that reported a majority of MM patients with fatigue had impaired independence and increased reliance on others.^42^ Interestingly, more subjects with muscle fatigue described “adapting” in the *definition* of fatigue as compared to its *impact*, and no subjects with muscle fatigue mentioned “adapting” in relation to their desired *improvement*, likely because “adapting” is an ability that they have already acquired.

## Imbalance

Falls were most frequently mentioned across the *definition*, *impact*, and desired *improvement* questions in those with imbalance (*n* = 19/33). Indeed, in the overall cohort, there was a significantly higher mention of falls across subjects (25/33 subjects) as compared to those who did not report falls (8/33), *p* < 0.001, Fisher’s exact test. Subjects with imbalance also referred to bathing, hobbies, and walking. Dressing and socializing were not discussed at all in relation to imbalance. No “other organ system involvement” was commonly mentioned in those with imbalance. In the general population, falls are typically a concern in aging and associated with a decline in function.^43^ Patients with a related genetic disorer, Friedreich Ataxia, have unsteady gait particularly on uneven terrain or in poor light, with increasing dependence on aids to walk.^44^ Indeed, imbalance was among the top five symptoms reported in PMD^4^ and is frequently captured on objective testing of patients with MM.^27^

## Peripheral neuropathy

In contrast, only three themes including school, shopping, and walking emerged in discussions of the *definition* of neuropathy, each only mentioned by one individual. Pain, mood, and hearing were discussed in terms of the *impact* of neuropathy. For pain, this theme was recurrently mentioned in patients with exercise intolerance and neuropathy, in comparison to muscle weakness, fatigue, and imbalance. Themes centered on “circumstances” and “independence and relationships” were not noted in response to the *definition* of neuropathy. “Adapting” was not noted in subjects with neuropathy. In terms of “other organ system involvement,” two-thirds of the cohort discussed pain in their *definition* of neuropathy. This contrasts with the *definition* of muscle fatigue, where cognition was commonly noted by 4/31 subjects (12.9%) with muscle fatigue, followed by vision and voice by 3/31 (9.7% each).

These subject narratives highlight that although key MM symptoms are separate entities with variable prominence across MM individuals,^27^ they are not mutually exclusive.

## Clinical trials

When asked if each MM symptom would motivate participation in a clinical trial, all subjects with exercise intolerance, 96% of subjects with muscle weakness, 90% with muscle fatigue, 84% with imbalance, and 73% with peripheral neuropathy stated that they would participate if this symptom were to be improved. This demonstrates the profound burden of myopathy symptoms, as reflected by the high subject motivation to participate in a clinical trial to treat their symptoms.

## Study limitations

We recognize that the study cohort of 33 subjects was limited, however, the depth and breadth of data yielded from only 33 MM structured and semi-structured, open-ended interviews was substantive as evidenced by the abundance of data presented and that data saturation was reached. In this study cohort, those subjects with peripheral neuropathy were underrepresented, which likely influenced the less elaborate discussions on the definition and impact of neuropathy. Further, symptoms of peripheral neuropathy, for instance decreased fine motor dexterity, distal muscle weakness in the hands and feet, and imbalance, overlap with other MM domains and thus may have been addressed in their responses to the imbalance and muscle weakness questions. For example, one subject noted difficulty inserting contact lenses in their eye due to muscle weakness and poor dexterity, on discussion of adapting to muscle weakness. Future qualitative studies of larger MM cohorts should be conducted to obtain a deeper understanding that is fully reflective of the breadth of multidimensional MM domains.

## Incorporating the MM patient perspective

FDA guidance calls for inclusion of the patient voice in the selection of outcome measures, clinical trial design, and the drug approval process.^45^ In this study, MM subject responses clearly emphasized the multidimensional impact of having an MM diagnosis, and demonstrated the broad and considerable impact of muscle weakness, muscle fatigue, exercise intolerance, imbalance, and peripheral neuropathy (Tables 3 and 4), thus underlining the critical need for MM-specific outcome measures to quantify symptoms across these key domains of MM in order to be clinically-meaningful.^27^ More noteworthy was the emerging significance of impaired dexterity in MM as was highlighted in subject responses discussing significant challenges with writing/typing for work and school, bathing (washing face, shaving), grooming hair, dressing (buttons, tying shoes, inserting contact lenses), eating and prepping food, and driving (turning the car key, pushing console buttons). Impaired dexterity in MM may be related to several factors including peripheral neuropathy, poor coordination from central nervous system defects (cerebellar ataxia, tremors, movement disorders, dystonia), and poor vision.^5,46^ Consequently, objective assessments of dexterity were included in the final development of the MM-Composite Assessment Tool (MM-COAST)^27^ as a quantitative measure of MM impairments for future clinical trials; in addition to objective assessments of muscle weakness, muscle fatigue, exercise intolerance, and imbalance. Indeed, results of this qualitative interview study provides content validity for the MM-COAST,^27^ and informed development of additional MM-specific outcome measures, the MM-Function Scale (Flickinger et al., unpublished), and the MM-IMPACT patient-reported outcome measure (Flickinger et al., unpublished). Lastly, the variable prominence of symptoms across MM domains in each individual was highlighted across subject responses, as was observed on objective MM-COAST assessments by Flickinger et al.^27^

In summary, our results demonstrate the substantial and widespread impact of MM across many aspects of MM patient’s lives, including the impact of each individual MM domain on ADLs; the need to adapt in order to preserve independence and QOL; impaired self-perception, participation in social activities, hobbies, and relationships; and their changing circumstances over time. These meaningful insights highlight the critical and emergent need for approved drug treatment(s) in this profoundly burdened patient population and will serve as a comprehensive resource to inform the physician, patient, industry and advocacy communities on outcome measure selection and clinical trial design; and to help inform regulatory agencies in the US FDA drug approval process for MM.

## Supplemental Material

sj-docx-10-taj-10.1177_20406223251344763 – Supplemental material for The profound implications of mitochondrial myopathy on activities of daily living: an observational qualitative study of standardized structured and semi-structured patient interviews Supplemental material, sj-docx-10-taj-10.1177_20406223251344763 for The profound implications of mitochondrial myopathy on activities of daily living: an observational qualitative study of standardized structured and semi-structured patient interviews by Elizabeth M. McCormick, James T. Peterson, Joaquim Diego D. Santos, Jean Flickinger, Rui Xiao, Richard Haas and Zarazuela Zolkipli-Cunningham in Therapeutic Advances in Chronic Disease

sj-docx-3-taj-10.1177_20406223251344763 – Supplemental material for The profound implications of mitochondrial myopathy on activities of daily living: an observational qualitative study of standardized structured and semi-structured patient interviews Supplemental material, sj-docx-3-taj-10.1177_20406223251344763 for The profound implications of mitochondrial myopathy on activities of daily living: an observational qualitative study of standardized structured and semi-structured patient interviews by Elizabeth M. McCormick, James T. Peterson, Joaquim Diego D. Santos, Jean Flickinger, Rui Xiao, Richard Haas and Zarazuela Zolkipli-Cunningham in Therapeutic Advances in Chronic Disease

sj-docx-4-taj-10.1177_20406223251344763 – Supplemental material for The profound implications of mitochondrial myopathy on activities of daily living: an observational qualitative study of standardized structured and semi-structured patient interviews Supplemental material, sj-docx-4-taj-10.1177_20406223251344763 for The profound implications of mitochondrial myopathy on activities of daily living: an observational qualitative study of standardized structured and semi-structured patient interviews by Elizabeth M. McCormick, James T. Peterson, Joaquim Diego D. Santos, Jean Flickinger, Rui Xiao, Richard Haas and Zarazuela Zolkipli-Cunningham in Therapeutic Advances in Chronic Disease

sj-docx-5-taj-10.1177_20406223251344763 – Supplemental material for The profound implications of mitochondrial myopathy on activities of daily living: an observational qualitative study of standardized structured and semi-structured patient interviews Supplemental material, sj-docx-5-taj-10.1177_20406223251344763 for The profound implications of mitochondrial myopathy on activities of daily living: an observational qualitative study of standardized structured and semi-structured patient interviews by Elizabeth M. McCormick, James T. Peterson, Joaquim Diego D. Santos, Jean Flickinger, Rui Xiao, Richard Haas and Zarazuela Zolkipli-Cunningham in Therapeutic Advances in Chronic Disease

sj-docx-6-taj-10.1177_20406223251344763 – Supplemental material for The profound implications of mitochondrial myopathy on activities of daily living: an observational qualitative study of standardized structured and semi-structured patient interviews Supplemental material, sj-docx-6-taj-10.1177_20406223251344763 for The profound implications of mitochondrial myopathy on activities of daily living: an observational qualitative study of standardized structured and semi-structured patient interviews by Elizabeth M. McCormick, James T. Peterson, Joaquim Diego D. Santos, Jean Flickinger, Rui Xiao, Richard Haas and Zarazuela Zolkipli-Cunningham in Therapeutic Advances in Chronic Disease

sj-docx-7-taj-10.1177_20406223251344763 – Supplemental material for The profound implications of mitochondrial myopathy on activities of daily living: an observational qualitative study of standardized structured and semi-structured patient interviews Supplemental material, sj-docx-7-taj-10.1177_20406223251344763 for The profound implications of mitochondrial myopathy on activities of daily living: an observational qualitative study of standardized structured and semi-structured patient interviews by Elizabeth M. McCormick, James T. Peterson, Joaquim Diego D. Santos, Jean Flickinger, Rui Xiao, Richard Haas and Zarazuela Zolkipli-Cunningham in Therapeutic Advances in Chronic Disease

sj-docx-8-taj-10.1177_20406223251344763 – Supplemental material for The profound implications of mitochondrial myopathy on activities of daily living: an observational qualitative study of standardized structured and semi-structured patient interviews Supplemental material, sj-docx-8-taj-10.1177_20406223251344763 for The profound implications of mitochondrial myopathy on activities of daily living: an observational qualitative study of standardized structured and semi-structured patient interviews by Elizabeth M. McCormick, James T. Peterson, Joaquim Diego D. Santos, Jean Flickinger, Rui Xiao, Richard Haas and Zarazuela Zolkipli-Cunningham in Therapeutic Advances in Chronic Disease

sj-docx-9-taj-10.1177_20406223251344763 – Supplemental material for The profound implications of mitochondrial myopathy on activities of daily living: an observational qualitative study of standardized structured and semi-structured patient interviews Supplemental material, sj-docx-9-taj-10.1177_20406223251344763 for The profound implications of mitochondrial myopathy on activities of daily living: an observational qualitative study of standardized structured and semi-structured patient interviews by Elizabeth M. McCormick, James T. Peterson, Joaquim Diego D. Santos, Jean Flickinger, Rui Xiao, Richard Haas and Zarazuela Zolkipli-Cunningham in Therapeutic Advances in Chronic Disease

sj-pdf-1-taj-10.1177_20406223251344763 – Supplemental material for The profound implications of mitochondrial myopathy on activities of daily living: an observational qualitative study of standardized structured and semi-structured patient interviews Supplemental material, sj-pdf-1-taj-10.1177_20406223251344763 for The profound implications of mitochondrial myopathy on activities of daily living: an observational qualitative study of standardized structured and semi-structured patient interviews by Elizabeth M. McCormick, James T. Peterson, Joaquim Diego D. Santos, Jean Flickinger, Rui Xiao, Richard Haas and Zarazuela Zolkipli-Cunningham in Therapeutic Advances in Chronic Disease

sj-pdf-2-taj-10.1177_20406223251344763 – Supplemental material for The profound implications of mitochondrial myopathy on activities of daily living: an observational qualitative study of standardized structured and semi-structured patient interviews Supplemental material, sj-pdf-2-taj-10.1177_20406223251344763 for The profound implications of mitochondrial myopathy on activities of daily living: an observational qualitative study of standardized structured and semi-structured patient interviews by Elizabeth M. McCormick, James T. Peterson, Joaquim Diego D. Santos, Jean Flickinger, Rui Xiao, Richard Haas and Zarazuela Zolkipli-Cunningham in Therapeutic Advances in Chronic Disease

## Declarations

**Ethics approval and consent to participate:** This study was approved by the CHOP IRB. All research activities complied with ethical regulations and were performed in accordance with CHOP regulations. All subjects or legally authorized representative(s) of subjects (minors or those with diminished capacity) completed the informed consent process and were enrolled into the CHOP IRB-approved research study #16-013364 (PI, ZZ-C) after providing written informed consent.

**Consent for publication:** All subjects and legally authorized representative(s) of subjects have consented to publication as this is discussed in the written informed consent process.

**Availability of data and materials:** All deidentified data supporting the findings of this study are available within the paper and its Supplemental Material. Additionally, deidentified data are available from the corresponding author upon reasonable request and with permission from The Children’s Hospital of Philadelphia Institutional Review Board.
