# Supplementary material for: The profound implications of mitochondrial myopathy on activities of daily living: an observational qualitative study of standardized structured and semi-structured patient interviews
Source: Ther Adv Chronic Dis. 2025 Jul 25;16:20406223251344763. doi: 10.1177/20406223251344763 (PMC12304646; doi:10.1177/20406223251344763)
Supplement: sj-docx-10-taj-10.1177_20406223251344763 – Supplemental material for The profound implications of mitochondrial myopathy on activities of daily living: an observational qualitative study of standardized structured and semi-structured patient interviews [file sj-docx-10-taj-10.1177_20406223251344763.docx]

| **Supplemental Table 3. Ranking of tasks in subjects with muscle weakness from easiest to hardest**  **(in ascending order from lowermost to the top)** |
| --- |
| Getting dressed |
| Putting on shirt |
| Wash/brush/style hair |
| Standing while waiting for the bus |
| Standing in the shower |
| Standing while cooking |
| Face washing |
| Eating |
| Climbing into the car |
| Scratching head |
| Reaching for remote |
