# Supplementary material for: The profound implications of mitochondrial myopathy on activities of daily living: an observational qualitative study of standardized structured and semi-structured patient interviews
Source: Ther Adv Chronic Dis. 2025 Jul 25;16:20406223251344763. doi: 10.1177/20406223251344763 (PMC12304646; doi:10.1177/20406223251344763)
Supplement: sj-docx-4-taj-10.1177_20406223251344763 – Supplemental material for The profound implications of mitochondrial myopathy on activities of daily living: an observational qualitative study of standardized structured and semi-structured patient interviews [file sj-docx-4-taj-10.1177_20406223251344763.docx]

**Semi-structured interview (all domains)**

1. What aspects of your daily life should be addressed in a clinical trial?
2. Do you experience symptoms of fatigue? (*If yes, see a-d below. If no, move to 3.)*
   1. What are the main symptoms of fatigue that you experience?
   2. What do these symptoms of fatigue mean to you and how do they impact your daily life?
   3. What would a slight improvement for your worst symptoms of fatigue look like to you?
   4. If we were able to improve this symptom to the extent that you just described, would you be willing to participate in a clinical trial?
3. Do you experience symptoms of exercise intolerance? *(If yes, see a-d below. If no, move to 4.)*
   1. What are the main symptoms of exercise intolerance that you experience?
   2. What do these symptoms of exercise intolerance mean to you and how do they impact your daily life?
   3. What would a slight improvement of your worst symptom of exercise intolerance look like to you?
   4. If we were able to improve this symptom to the extent that you just described, would you be willing to participate in a clinical trial?
4. Do you experience symptoms of muscle weakness? *(If yes, see a-t below. If no, move to 5.)*
   1. Are you weaker in your arms or your legs? **Response options: Arms, Legs, Both**
   2. Are you able to walk? **Response options: Yes, No**
   3. Are you able to stand without support? **Response options: Yes, No**
   4. Do you experience muscle weakness when standing while waiting for the bus? **Response options: Yes, No**
   5. Do you experience muscle weakness when standing while cooking? **Response options: Yes, No**
   6. Do you experience muscle weakness when getting dressed? **Response options: Yes, No**
   7. *For men only:* Do you experience muscle weakness when using the toilet standing? **Response options: Yes, No**
   8. Do you experience muscle weakness when showering standing up? **Response options: Yes, No**
   9. Do you experience muscle weakness when climbing into a car? **Response options: Yes, No**
   10. Are you able to reach your arms up above your head? **Response options: Yes, No**
   11. Are you able to reach your arms out in front of you? **Response options: Yes, No**
   12. Do you feel arm muscle weakness when you scratch your head? **Response options: Yes, No**
   13. Do you feel arm muscle weakness when you are washing, brushing, and/or styling your hair? **Response options: Yes, No**
   14. Do you feel arm muscle weakness when you are putting on your shirt? **Response options: Yes, No**
   15. Do you feel arm muscle weakness when you are reaching for the remote? **Response options: Yes, No**
   16. Do you feel arm muscle weakness when you are washing your face? **Response options: Yes, No**
   17. Do you feel arm muscle weakness when you are eating? **Response options: Yes, No**
   18. Added comments about main symptoms of muscle weakness:
   19. What would a slight improvement of your worst symptom of muscle weakness look like to you?
   20. If we were able to improve this symptom to the extent you just described, would you be willing to participate in a clinical trial?
5. Do you experience symptoms of imbalance? *(If yes, see a-d below. If no, move to 6.)*
   1. What are the main symptoms of imbalance that you experience?
   2. What do these symptoms of imbalance mean to you and how do they impact your daily life?
   3. What would a slight improvement of your worst symptom of imbalance look like to you?
   4. If we were able to improve this symptom to the extent that you just described, would you be willing to participate in a clinical trial?
6. Do you experience neuropathy? *(If yes, see a-d below. If no, move to 7.)*
   1. What are the main symptoms of neuropathy that you experience?
   2. What do these symptoms of neuropathy mean to you and how do they impact your daily life?
   3. What would a slight improvement of your worst symptom of neuropathy look like to you?
   4. If we were able to improve this symptom to the extent that you just described, would you be willing to participate in a clinical trial?
7. Are there any other symptoms that you commonly experience that we have not discussed? *(If yes, see a-d below. If no, move to 8.)*
   1. What does {symptom listed by subject} mean to you and your daily life?
   2. What would a slight improvement of your worst symptom of {symptom listed by subject} look like to you?
   3. What would a slight improvement of your worst symptom of {symptom listed by subject} look like to you?
   4. If we were able to improve this symptom to the extent that you just described, would you be willing to participate in a clinical trial?
8. Did answering these questions impact the way you feel about your current state?
9. Any other comments?
