# Supplementary material for: The profound implications of mitochondrial myopathy on activities of daily living: an observational qualitative study of standardized structured and semi-structured patient interviews
Source: Ther Adv Chronic Dis. 2025 Jul 25;16:20406223251344763. doi: 10.1177/20406223251344763 (PMC12304646; doi:10.1177/20406223251344763)
Supplement: sj-docx-5-taj-10.1177_20406223251344763 – Supplemental material for The profound implications of mitochondrial myopathy on activities of daily living: an observational qualitative study of standardized structured and semi-structured patient interviews [file sj-docx-5-taj-10.1177_20406223251344763.docx]

| **Theme** | **Description** |
| --- | --- |
| **Activities of daily living** | |
| Bathing | Any mention of bathing, showering, washing hair |
| Chores | Any mention of household chores |
| Dressing | Any mention of getting dressed, including hair styling or shaving |
| Driving | Any mention of driving or being a passenger in a car, any mention of cars or parking |
| Eating | Any reference to eating including cooking/preparing food |
| Hobby | Any mention of a hobby; could be a sport, leisure activity, or play |
| School | Any mention of school or classes, including getting through the day and getting to school, learning |
| Shopping | Any mention of shopping (such as grocery, clothes) including getting into store and navigating around store |
| Sleep | Any mention of sleep or sleeping, naps |
| Socializing | Any mention of socializing including playing with friends, leisure activities with friends/family |
| Stairs | Any mention of climbing stairs, getting up/down stairs |
| Therapies | Any reference to services such as physical, occupational, and/or speech therapies |
| Walking | Any mention of walking, ambulation |
| Work | Any mention of work, including work day and desk/computer |
| **Adapting to life with MM** | |
| Adapting | Any reference to how subject has adapted to the situation, or how they make the current situation work |
| Need to take a break | Any mention of needing to take a break |
| Planning ahead | Any mention of needing to plan in the future, whether planning for the day or planning over time |
| Pushing past my limits | Any mention of pushing themselves, pushing past something, over-doing it |
| **Circumstances** | |
| Current ability | Any mention of what current ability is |
| Doing what I want | Any mention of doing activities or other tasks they want or need to do |
| What I used to do | Any mention of something they did in the past, or activities they were able to do in the past |
| **Independence and relationships** | |
| Communication | Any mention of communication with others; communication devices, conversations, talking |
| Doctors | Any mention of doctors, doctors’ offices |
| Family | Any mention of family, family members |
| Comparison to others | Any comparison or mention made to others |
| Dependence | Any mention of dependence or reliance such as on other people, assistive devices, and/or medicines |
| **Other organ system involvement** | |
| Cognition | Any mention of memory, processing, mental fatigue |
| GI | Any mention of gastrointestinal tract and associated issues (such as nausea, diarrhea, constipation) |
| Hearing | Any mention of hearing, including hearing ability and/or loss |
| Mood | Any mention of mood, emotions, feelings |
| Outward appearance | Any mention of their appearance, how others perceive them |
| Pain | Any mention of pain, discomfort, cramping |
| Ptosis | Any mention of ptosis |
| Reproductive | Any mention of reproduction, family planning |
| Respiratory | Any mention of respiratory involvement, becoming winded, breathing |
| Vision | Any mention of vision, reading |
| Voice | Any mention of voice, speaking |
| **Severity** | |
| Minor | Any mention of something being minor, not that bad, slight |
| Severe | Any mention of something being severe, bad, persistent, chronic |
| **Other** | |
| Falls | Any mention of falling or falls |
| Worry | Any mention of a worry they had or are having, concern, anxiety, consideration for the future |
