# Supplementary material for: The profound implications of mitochondrial myopathy on activities of daily living: an observational qualitative study of standardized structured and semi-structured patient interviews
Source: Ther Adv Chronic Dis. 2025 Jul 25;16:20406223251344763. doi: 10.1177/20406223251344763 (PMC12304646; doi:10.1177/20406223251344763)
Supplement: sj-docx-6-taj-10.1177_20406223251344763 – Supplemental material for The profound implications of mitochondrial myopathy on activities of daily living: an observational qualitative study of standardized structured and semi-structured patient interviews [file sj-docx-6-taj-10.1177_20406223251344763.docx]

***Themes identified***

Subjects commonly *defined* their muscle weakness, exercise intolerance, muscle fatigue, imbalance, and peripheral neuropathy symptoms, the *impact* of these symptoms, and what a slight *improvement* would be in the context of ADLs. Indeed, bathing, chores, dressing, driving, eating, sleeping, and walking commonly arose in discussion, as well as hobbies, school, shopping, socializing, therapies, and work. Responses not only centered on the individual but also on activities they enjoyed and/or their environment. Notably, some ADLs that were mentioned are critical to managing their health, including shopping for groceries and participating in physical, speech, and occupational therapies. Subjects also described GI symptoms, pain, and their outward appearance, consistent with studies in other neuromuscular disorders^28^. This emphasizes the broad, systemic impact of having a MM diagnosis.

Worry was mentioned in the context of anticipating the negative repercussions of their symptoms, for instance, concern for not being able to keep up with their children or being unable to participate in activities with long term health benefits, such as exercise. These concerns were clearly of distinct significance across the MM study cohort. It is worth noting that these concerns may not be readily volunteered by MM patients at their routine clinic visits unless specifically prompted by their physician, hence MM patient worries and fears are likely under-recognized.

Similarly, ‘falls’ was mentioned across several MM key domains and by more than 75% of the study subjects without prompt, highlighting the high prevalence of ‘falls risk’ across this MM cohort. In the general population, falls are typically a concern in aging and associated with a decline in function^42^. Patients with a related genetic disorder, Friedreich Ataxia, have unsteady gait particularly on uneven terrain or in poor light, with increasing dependence on aids to walk^43^. Indeed, imbalance was among the top five symptoms reported in PMD^4^ and is frequently captured on objective testing of patients with MM^27^.

Subjects deliberated on topics related to ‘adapting to life with MM’, including the necessity of ‘planning ahead,’ the ‘need to take a break,’ and the need to consider ‘pushing past [their] limits.’ The most frequently reported examples of ‘adapting’ specifically were in relation to eating, including resting an arm while eating and use of adaptive utensils. Individuals with MM may not emphasize the substantial impact of MM on daily life in routine discussions, because they have learned to adapt. The ability to adapt by ‘planning ahead’, ‘need to take a break’ and the ‘need to push past their limits’ to ensure daily ADLs were achieved should not be misinterpreted as the individual having low disease burden. Pushing past their limits can be followed by a negative impact (such as exacerbated muscle fatigue and pain) for a prolonged period of time. Given the fluctuations and often progressive nature of MM, the need to adapt is a continuous and lifelong process. Achieving successful adaptation at one point in time in their disease phase may not be sustainable over time. Indeed, some subjects referred to a prior ability to adapt until they were no longer able.

Subjects also spoke about themes related to their circumstance including ‘current abilities’, doing what they desired (‘doing what I want’), and what they were able to do in the past (‘what I used to do”). Several subjects focused on what they used to be able to do and voiced a desire for prior abilities to be restored. In this context, subjects expressed that their symptoms prevented them from ‘doing what they wanted to do’. As such, the relevance of ‘treatment’ to these subjects was the desire to return to a prior level of functioning when they were still able to adapt to their physical limitations.

Several subjects volunteered that their communication was impaired. Communication is a fundamental skill in promoting quality of life, including social interaction to feel connected to others at home and in the community. For example, subjects described physical limitations to communication (e.g., as one subject described in her son “*His disease is so involved that his ability to use his communication device is limited*,” and another parent noted “*His speech is almost gone and he tried very hard to communicate verbally but people cannot understand him*”), but also described cognitive barriers to communication (e. g. one subject noted, “*[MM] slows me down cognitively. I can’t follow a long, in-depth conversation*,” while another noted, “*The mental exercise of trying to interact with a bunch of people… hard to explain how exhausting it is*”).

Themes of dependence and external relationships also arose without prompt, including ‘dependence’ on other individuals and/or in ‘comparison to others.’ Some subjects described their symptoms in ‘comparison to others’, or by comparing themselves to family members or to healthier individuals. The perception of ‘severity’ was repeatedly discussed and, at times, in relation to others. Families with multiple affected individuals may have distinct perceptions of their MM symptoms as compared to a single affected individual in a family of healthy individuals. Indeed, an affected MM individual with multiple affected family members may perceive ‘severity’ very differently due to prior experience of an affected family member(s) who succumbed to their MM diagnosis.
