# Supplementary material for: The profound implications of mitochondrial myopathy on activities of daily living: an observational qualitative study of standardized structured and semi-structured patient interviews
Source: Ther Adv Chronic Dis. 2025 Jul 25;16:20406223251344763. doi: 10.1177/20406223251344763 (PMC12304646; doi:10.1177/20406223251344763)
Supplement: sj-docx-7-taj-10.1177_20406223251344763 – Supplemental material for The profound implications of mitochondrial myopathy on activities of daily living: an observational qualitative study of standardized structured and semi-structured patient interviews [file sj-docx-7-taj-10.1177_20406223251344763.docx]

***MM characterization***

**Definition of MM**

When asked about their main symptoms of each domain, “What are the main symptoms of [muscle fatigue, exercise intolerance, imbalance, neuropathy] that you experience?” subjects defined their symptoms by elaborating on their ADLs, highlighting the profound and broad impact of MM symptoms on daily life. School and walking were mentioned across all MM domains of muscle fatigue, exercise intolerance, imbalance, and neuropathy. Walking was noted by more than 30% of subjects with exercise intolerance and imbalance. Bathing and hobbies were mentioned across all MM domains except neuropathy. Socializing and therapies were only mentioned in relation to exercise intolerance and fatigue. Dressing was not used to describe the definition of any domains. Most subjects defined their manifestations by their ‘current ability’, while others defined their symptoms as ‘what they used to be able to do’ except in neuropathy.

Subjects also brought up ‘other organ system involvement’ when describing their MM symptoms across all domains except for imbalance. Pain was mentioned across all domains, and by multiple subjects with neuropathy.

Themes grouped under ‘adapting to life with MM’ were frequently mentioned in those with exercise intolerance and fatigue, as compared to those subjects with imbalance and neuropathy. ‘Pushing past limits’ was mentioned across all MM domains. ‘Adapting’ was specifically noted by subjects with exercise intolerance, fatigue, and imbalance.

In terms of themes of circumstance, subjects with exercise intolerance, fatigue, and imbalance more frequently defined their symptoms by their ‘current ability’ (12/29, 41.4%; 17/31, 54.8%; and 6/19, 31.6% respectively), compared to the context of ‘what they used to be able to do’ (2/29, 6.9%; 2/31, 6.5%, and 1/19, 5.3%, respectively). ‘Needing to take a break’ was highlighted when discussing exercise intolerance and fatigue. Almost a quarter of all subjects with exercise intolerance and fatigue defined their symptoms as keeping them from doing what they wanted.

Themes centered around ‘independence and relationships’ also arose in response to questions regarding defining MM. Indeed, 16.1% (5/31) of those with fatigue mentioned communication and 6.5% (2/31) mentioned family. This contrasts with imbalance, where communication and family were not mentioned. Alternatively, 26.3% (5/19) of those with imbalance defined this symptom by describing their ‘dependence’ on external sources. Subjects with neuropathy did not discuss any themes in relation to ‘independence and relationships’.

Exercise, fatigue, and neuropathy were all defined as ‘severe’ by at least one subject. No one with exercise intolerance implied that this symptom was minor. No individuals with exercise intolerance or fatigue discussed ‘worry’ in their definition however more than 15% of those with imbalance did.

**Impact of MM**

Subjects notably elaborated more on the impact of their symptoms as compared to when asked to define their symptoms. The narratives on “What do these symptoms of [muscle fatigue, exercise intolerance, imbalance, neuropathy] mean to you and how do they impact your daily life?” or “What other comments do you have on your symptoms of muscle weakness?” and “What would a slight improvement of your worst symptom of [muscle fatigue, exercise intolerance, muscle weakness, imbalance, neuropathy] look like to you?” were lengthy and informative, highlighting the importance of how questions are posed to study subjects with MM.

Bathing, driving, school, and walking were impacted across all five MM domains, exemplifying the impact of MM on almost every daily task. Chores, eating, hobbies, shopping, and stairs were impacted across every MM domain except neuropathy, while some ADLs were impacted in specific domains, such as ‘work’ arising in relation to exercise intolerance and fatigue, with 25.8% (8/31) of subjects with fatigue reporting work to be affected.

Pain was mentioned in relation to impact in every MM domain except fatigue. Almost 10% of those with muscle weakness mentioned ‘outward appearance’, whereas this was only mentioned by one person with fatigue and imbalance, but in five people with muscle weakness.

‘Adapting’ specifically and ‘doing what they wanted’ was impacted across every MM domain. The ‘need to plan ahead’ was impacted in every MM domain except muscle weakness and neuropathy, and ‘need to take a break’ was impacted in exercise intolerance and fatigue.

‘Dependence’ was noted as an impact across every domain. Moreover, subjects in all domains besides neuropathy described dependence in relation to other individuals. Family was impacted in exercise intolerance, fatigue, and imbalance domains. Communication was impacted in 19.4% (6/31) of those with fatigue.

No subject with fatigue described its impact as ‘severe’ compared to almost 20% of those with muscle weakness. This implies that fatigue may not be severe or acute, but rather pervasive across all the ADLs. ‘Worry and apprehension’ were mentioned or implied in every MM domain except for neuropathy with the strongest recurrence when discussing impact of exercise intolerance.

Falls were mentioned in each domain, with almost half of those with imbalance discussing falls as impactful in their life.

**Slight improvement in MM symptoms**

No themes were mentioned more than once when subjects with neuropathy were asked what a slight improvement would look like. Subjects discussed slight improvements in chores, hobbies, and walking in the domains of muscle weakness, exercise intolerance, fatigue, and imbalance. Improvements in dressing and socializing were mentioned by those with muscle weakness, exercise intolerance, and fatigue only. Improvement in bathing was mentioned in relation to exercise intolerance, fatigue, and imbalance but not muscle weakness and neuropathy. Cognition was noted as a desired area of improvement in those with exercise intolerance, fatigue, and imbalance.

Themes of circumstance, including ‘doing what I want’ was mentioned by 34.5% (10/29) of those with exercise intolerance, 19.5% (6/31) with fatigue, 15.8% (3/19) with imbalance, and 7.4% (2/27) with muscle weakness, highlighting how limiting exercise intolerance and fatigue can affect individuals. Needing to take a break was mentioned in between 11-14% of those with exercise intolerance, fatigue, and muscle weakness.

Dependence on other people or assistive devices was the theme most commonly mentioned when asked ‘what a slight improvement would look like’. Indeed, 21.1% (4/19) of those with imbalance, 14.8% (4/27) of those with muscle weakness, 9.7% (3/31) of those with fatigue, and 6.9% (2/29) of those with exercise intolerance discussed this.
