# Supplementary material for: The profound implications of mitochondrial myopathy on activities of daily living: an observational qualitative study of standardized structured and semi-structured patient interviews
Source: Ther Adv Chronic Dis. 2025 Jul 25;16:20406223251344763. doi: 10.1177/20406223251344763 (PMC12304646; doi:10.1177/20406223251344763)
Supplement: sj-docx-8-taj-10.1177_20406223251344763 – Supplemental material for The profound implications of mitochondrial myopathy on activities of daily living: an observational qualitative study of standardized structured and semi-structured patient interviews [file sj-docx-8-taj-10.1177_20406223251344763.docx]

| **Muscle weakness** | |
| --- | --- |
| **Impact** | - “Shaving can be really difficult, and I'm sure that's the same for females shaving their legs. I will only shave every 3 days.” - "I've done laundry my whole life but now it exhausts me to lift wet clothes out of the washer. I used to lift a lot at once, and now I have to lift a piece or two at a time, but that repetitive movement then makes my arms tired, as well." - "He cannot lift a glass of water to his mouth, he has to use a straw. When he is too tired, muscles too weak, even the way he drives his wheelchair is dangerous. His arms are so weak right now that I have to feed him, he will take one or two bites and then become too weak. He can't dress and undress himself because his muscles are too weak." - "Even like putting in my contacts, I don't have the strength and dexterity to put my contact into my eye. I have to rest my arm on the counter and lower my head to my hand to put in a contact. Simple things that seem so easy, I am not able to do. When I eat, I am always resting my forearm on the table to stabilize my arm and lower my head to my hand to take off some of the weight." - "One of the things that has changed for me is that I don't worry about showering every day or styling my hair. I need to put my energy into the household things like doing the dishes and taking care of the laundry." - "For my arms, simple things like pouring a pot of coffee. I can only pour a cup of coffee if the pot is half full. Or picking up a half gallon of juice at the grocery store and trying to read the label... Even if I go to the coffee shop and want to carry a full cup of coffee, I struggle to carry it to the table" - “When you were talking about climbing into a car- we actually had a higher up SUV and he would need to kneel down then climb in. We sold that car and got a lower car with buttons that open and close the doors because we felt that it wasn't good for his self esteem, even though he could get into the different car.” - “His disease is so involved that his ability to use his communication devices is limited. Weakness has affected all aspects of life for [him]. Feeding, dressing, using his computer, wheelchair, opening doors are all difficult tasks for [him] at this point.” - “I also have ptosis, and I'm not sure if that is considered muscle weakness- it bothers me a lot in how it affects my appearance. But it also obstructs my vision, which is just an annoyance.” |
| **Improvement** | - “If I could do more sorting, and I'm just using sorting as an example, or folding laundry, it would be better to have longer endurance with these types of tasks. I always have to stop- my arms feel weak and I have diaphragm issues. If I could go for longer, that would be much better. The same for walking. Being able to go for longer would be great.” - “Just not feeling like I have to sit on the stool in my bathroom or the kitchen. I have to sit on the stool even when I am brushing my teeth.” - “Being able to prepare a full meal on my own and then being able to eat afterwards.” - “…[his own ability] to take his very loose shirt off or on…these things would be slight but also a big improvement." - “For us, that would mean that he would be able to style his hair- he's a teenage boy, he wants to style it with mousse. And I would love to see him be able to zip his own coat and feed himself.” - "Being able to go about life, like just waiting for a bus- the bus stop I go to doesn't have a bench- so not having to deal with being tired from standing would be great." - “For him to be able to pick up a glass of water and bring the straw to his mouth. A slight improvement would be to grab his food on his own.” - "... it would be great if he could hold little toys in his hands." - "Being able to walk with a walker and have more endurance with the walker. He can use the walker now, but he can't go very far." |
| **Exercise intolerance** | |
| **Definition** | - “When I walk long distances, if I walk 5 minutes, my legs start burning and I have to sit down.” - “Standing for a long time. I can only stand for about 3-4 minutes without having to sit down. Standing still is horrible, I can't do it - my upper legs feel like they are burning.” - “When we're running in gym class, everyone is running nonstop and I have to sit down and take a drink, and can the maybe join back in…writing - if we are writing a big paragraph I have to stop and take a break after a few sentences.” |
| **Impact** | - “I can't exercise. Steps, walk to a restaurant, lifting weights, walking around a track. I am very disappointed I can't exercise like normal people - for good health, for goals, for something to do. It really affects my quality of life that I can't do normal exercise." - "Exercise intolerance limits me everyday from what I can do. Housework, yardwork, exercise to stay in shape - everyday it impacts my life. I can't do things I want to do." |
| **Improvement** | - “That would make a big difference for us, being able to finish a daily activity. We cancel a lot of stuff with [him] because he gets too tired. Sometimes we have plans two months in advance, like a field trip, but because he is too tired we wait and decide if he is too tired to participate.” - "If I could go walk around the block outside instead of holding onto the treadmill, that would really be wonderful." - “…to have a day and just do things but not feel it. Like take a walk and then take the dogs for another walk, not having to depend on someone else to do it.” |
| **Fatigue** | |
| **Definition** | - "There are days where I would wake up and it feels like I ran a marathon or I worked out really really hard. I wake up and my legs are just really sore and achy, it takes me awhile for that to go away." - "It's so hard to describe the fatigue. It isn't sleepiness. It feel like a combination of the flu and a hangover. It feels like this overwhelming tiredness just doing everyday ordinary activities. Mental fatigue also affects me. The mental exercise of trying to interact with a bunch of people, like out to dinner with friends. It should be fun and great to go out but it is…hard to explain how exhausting it is." - “There is general overall fatigue and then there is muscle fatigue. Feeling sluggish or tired. Sometimes I just feel sluggish or blah, I just don't feel like moving or doing anything which is unusual for me. I don't like just sitting on the couch doing nothing but sometimes I have to.” - “I went into [the grocery store] yesterday and the walk from a handicap parking space to the aisle, I had to stop. It is as if I need to recover and something tells me I can't go on, my body tells me to not keep going or I might fall over. I feel it in my arms and diaphragm fatiguing, a collection of symptoms that make me stop and recover.” |
| **Impact** | - “I am restricted from going anywhere big like a library or large stores. I have to check everything, like if there are too many steps. I rule out activities and errands if they are too far or too many steps. I won't do something if it is past my limit, like stairs.” - "I … need to plan for if I will be able to drive. I have to think, ‘Is this really worth it? Will I be able to get home?’ There have been times when I've had to pull over on the side of the road to take a break." - "It effects my life because I have to turn things down, like walking across the street to lunch. I say I can't go. [This] affects socializing and getting things done like errands." - “I can't work anymore. I haven't been able to work for awhile. I was full time and then part time in my 30s and now I can't work at all. I feel like that is a basic thing of being an adult and I can't do that.” - “I can no longer work. When I was working, I would work from home and need to take 2 or 3 naps throughout the day to get through it. That really impacted my life. The fatigue doesn't allow me to hold down a full-time job. When I used to work full time and live on my own, by the end of the day and trying to cook a meal, I would be so exhausted. Barely having the energy to cook a meal, I would just get takeout and eat junky food because it is easier.” - “It seems like if I do a lot for like three days, I will literally have a crash usually. If I push, push, push, and then I will crash. I can't even get out of bed. I feel like I will miss out on family time.” - “Completing normal household chores and getting done what I need to get done. I need to strategize my day, plan for things that I might not be able to get done. I can't make calls when I'm jumbling my words. People think I'm drunk or having a stroke, so you tend to work that into planning my day.” - "Work is definitely affected and any type of socializing or doing anything physical. Like thinking about wanting to go to the beach, I have to think about walking through the sand, changing into beach clothes, traveling there." |
| **Improvement** | - “It is so variable, some days I will have more energy than the days before. I would probably say getting ready in the morning - the process of getting up, showering, brushing teeth, combing hair, making breakfast - that is all really fatiguing. That would be something I would recognize every day, seeing or noticing an improvement." - “…right now, if I could be up one full day without taking rest periods. Freedom. If I could actually be up more throughout the day. Maybe take a vacation. For me, it would be like living. I have missed so much of my son's life, you feel like you miss so much life.” |
| **Imbalance** | |
| **Impact** | - “Even when seated we have to be careful about what kind of seat and what is around her. An example, the cafeteria at school has stools with no backs on them and she can't sit on that because she would fall off. They have to put padding on the table in front of her at school because she could fall forward and hit her face on the table. Virtually everything is affected by her imbalance it is so severe.” - “…he seems really strong, but the signal from his brain just can't get him to sit up or stand on his own. He can't walk, he can't stand, he can't go up stairs. He hasn't been upstairs in probably 15 years, if not longer. He's always in the wheelchair, that's his life." - “Walking in front of strangers at all, I won't do that. I'll hold my daughter's or husband's hand so I don't veer off left.” - “I worry that someone might think I am drunk walking down the street.” |
| **Improvement** | - "If he could coordinate and transfer himself into and out of bed, that would mean everything to him." - "Being able to stand up on his own and just hold a hand. Or being able to sit up longer and be able to play a game without assistance." - “Being able to get around and do daily activities without feeling it, without feeling like I was going to fall or topple. I would love to do activities without even having to think about balance.” - "…I know it would mean the world to him if he could do more for himself. If he could stand or walk, that would be a miracle." |
| **Neuropathy** | |
| **Impact** | - “Walking is definitely impacted since I don't have much feeling in my feet. Also standing, it's not really pain, just uncomfortable pressure from my legs down through my feet.” - “They don't impact my daily life as in limiting activities, but it is just another annoyance, thing on the list.” |
