# Supplementary material for: The profound implications of mitochondrial myopathy on activities of daily living: an observational qualitative study of standardized structured and semi-structured patient interviews
Source: Ther Adv Chronic Dis. 2025 Jul 25;16:20406223251344763. doi: 10.1177/20406223251344763 (PMC12304646; doi:10.1177/20406223251344763)
Supplement: sj-docx-9-taj-10.1177_20406223251344763 – Supplemental material for The profound implications of mitochondrial myopathy on activities of daily living: an observational qualitative study of standardized structured and semi-structured patient interviews [file sj-docx-9-taj-10.1177_20406223251344763.docx]

| **Supplemental Table 2. Ranking of ADLs from easiest to hardest**  **(in ascending order from lowermost to the top)** |
| --- |
| Climbing stairs |
| Walking |
| Hobbies |
| Chores |
| Shopping |
| Socializing |
| School |
| Therapies |
| Work |
| Dressing |
| Bathing |
| Driving |
| Eating |
| Sleep |
